# (C_2_G_4_)_n_ repeat expansion sequences from the *C9orf72* gene form an unusual DNA higher-order structure in the pH range of 5-6

**DOI:** 10.1371/journal.pone.0198418

**Published:** 2018-06-18

**Authors:** Prince Kumar Lat, Dipankar Sen

**Affiliations:** 1 Department of Molecular Biology & Biochemistry, Simon Fraser University, Burnaby, British Columbia, Canada; 2 Department of Chemistry, Simon Fraser University, Burnaby, British Columbia, Canada; University of Oklahoma, UNITED STATES

## Abstract

Massive expansion of a DNA hexanucleotide sequence repeat (C_2_G_4_) within the human *C9orf72* gene has been linked to a number of neurodegenerative diseases. In sodium or potassium salt solutions, single-stranded d(C_2_G_4_)_n_ DNAs fold to form G-quadruplexes. We have found that in magnesium or lithium salt solutions, especially under slightly acidic conditions, d(C_2_G_4_)_n_ oligonucleotides fold to form a distinctive higher order structure whose most striking feature is an “inverted” circular dichroism spectrum, which is distinguishable from the spectrum of the left handed DNA double-helix, Z-DNA. On the basis of CD spectroscopy, gel mobility as well as chemical protection analysis, we propose that this structure, which we call “iCD-DNA”, may be a left-handed Hoogsteen base-paired duplex, an unorthodox G-quadruplex/i-motif composite, or a non-canonical, “braided” DNA triplex. Given that iCD-DNA forms under slightly acidic solution conditions, we do not know at this point in time whether or not it forms within living cells.

## Introduction

The repeat expansion of a hexanucleotide DNA sequence (CCGGGG) found in the 5’-untranslated region of the *C9orf72* gene has been shown to be causally linked to Frontotemporal Lobar Dementia and familial Amyotrophic Lateral Sclerosis (FTD/ALS) [1, 2]. In normal individuals, the number of the hexanucleotide repeats in the *C9orf72* gene is ~20–30 or less [3]). When expansion extends to tens to thousands of repeats, it leads to pathology [4–6]. A conclusive understanding of the pathological role of the *C9orf72* expansion in the etiology of FTD/ALS remains to be established; however, three major mechanisms have so far been proposed [7]. At the level of DNA, the d(GGGGCC) repeat expansion single strand and its complementary strand have been shown in vitro to form unusual secondary structures, namely hairpin folds, G-quadruplexes, i-motif and R loops [8–10]. These unusual structures, if present in repeat-expansion afflicted neurons, can potentially cause down-regulation in gene expression leading to reduced levels of the coded protein [11–13]. Indeed, the repeat expansion has been shown to decrease c9orf72 expression [1]. The DNA as well the sense [r(GGGGCC)_n_] and antisense [r(GGCCCC)_n_] RNAs from this gene are capable of forming, variously, G-quadruplexes [8, 14–16], i-motifs [10] and other folds [17]. RNA foci arising from insoluble and tangled transcripts are seen in the nucleus and cytoplasm of repeat-expansion containing neurons [18–20]. The presence of such foci likely serve to sequester key cellular RNA-binding proteins (such as splicing factors) [21–24] as well as cellular heme [25]. At the level of protein, the r(GGGGCC)_n_ and r(CCCCGG)_n_ transcripts undergo non-AUG initiated translation to produce dipeptide repeat proteins (DPR): (GA)_n_, (GP)_n_ and (GR)_n_ (from the sense strand) and (PR)_n_ and (PA)_n_ (from antisense strand) [26–28]. These proteins accumulate in the brain and spinal cord of the C9orf72 mutation-carrying population [27, 29] and are also hypothesized to contribute to neurodegeneration.

In studying potential secondary structures formed by repeats of d(C_2_G_4_) single stranded DNA sequences, we found the formation of an unexpected higher-order structure in response to incubation at moderate to high DNA concentrations. Described below is a study that uses circular dichroism (CD), native gel mobility and footprinting analysis to investigate this unusual higher-order DNA structure.

## Materials and methods

### DNA preparation and incubation

All DNA oligonucleotides were purchased from the Core DNA Services Inc. (Calgary, Canada). Oligonucleotides were dissolved in TE buffer (10 mM Tris, 0.1 mM EDTA, pH 7.4), purified once by ethanol precipitation from TE containing 400 mM LiCl. DNA pellets so obtained were redissolved in TE buffer. Oligonucleotides used for native gel mobility analysis and for DMS footprinting experiments were 5' labeled with ^32^P using γ-^32^P ATP and a standard kinasing protocol, and then PAGE-purified following a pre-treatment with 10% (v/v) freshly prepared piperidine (v/v) at 90° C for 30 minutes prior to lyophilization.

For incubations, the DNA was heat denatured at 100° C in a water bath for 4 minutes, followed by immediate cooling in ice. Incubations were generally carried out with 700 μM DNA in the appropriate buffer solution, at 37° C. The DNA solution was then diluted with the same or another buffer to give 20 μM DNA, suitable for CD spectroscopy and other experiments. However, in many instances, incubations were carried out directly with 20 μM DNA, with end results indistinguishable from the higher concentration DNA incubations.

### Native PAGE electrophoresis and DMS protection assay

Native gel electrophoresis of d(C_2_G_4_)_7_ was carried out in 7.5% bis/polyacrylamide (29:1) gels and run in TAE-Li Buffer (20 mM Tris, 1mM EDTA, 45 mM acetic acid and 20 mM lithium citrate, pH 5.2). Gels were run at 22° C for 4 hrs at 9 W with efficient cooling. Incubated DNA solutions were mixed with native gel loading buffer (50 mM Tris acetate, pH 5.2, 30% glycerol and loading dyes) prior to loading on gels.

Following incubation of ^32^P-5’-labeled d(C_2_G_4_)_7_ at a concentration of 700 μM in 150 mM Lithium Citrate, pH 5.2, at 37°C for 14 hrs, partial DNA modification with dimethyl sulfate (DMS) was carried out by addition of 0.2% DMS (freshly prepared in 10 mM Tris, pH 5.2). The mixture was allowed to incubate for 30 min at 22° C, and the reaction stopped using ß-mercaptoethanol. Treated solutions were then run on native gels. The observed slow (s) and fast (f) moving bands were excised from the gel and their DNA eluted into TE buffer. DNA was recovered by ethanol precipitation, washed with cold 70% ethanol, dried, dissolved in freshly prepared 10% v/v piperidine, and heated at 90° C for 30 min. Following lyophilization, the DNA was dissolved in denaturing gel loading buffer (95% formamide, 1 mM EDTA, and loading dyes) and run in 20% denaturing/ sequencing gels.

### Gel data analysis

Imaging and densitometry of native and sequencing gels running ^32^P-labeled DNA were carried out on a Typhoon 9410 Phosphorimager (Amersham Biosciences). Quantitation was carried out using the ImageQuant 5.2 software (Amersham).

### Circular dichroism spectroscopy

Following incubation and dilution of the DNA, as above, CD spectra was recorded in a Jasco-810 Spectropolarimeter (Jasco, Easton, MD) using a quartz cell of 0.5 mm optical path length. The scanning speed was fixed at 500 nm/min, with a response time of 1 s, and scans were carried out over a wavelength range of 220–320 nm. The spectra in the figures represent averages of 5 sequential scans, all measured at 22° C with baseline correction.

## Results and discussion

### Inverted CD spectrum of d(C_2_G_4_)_7_ in the absence of G-quadruplex stabilizing cations

**Fig 1:**
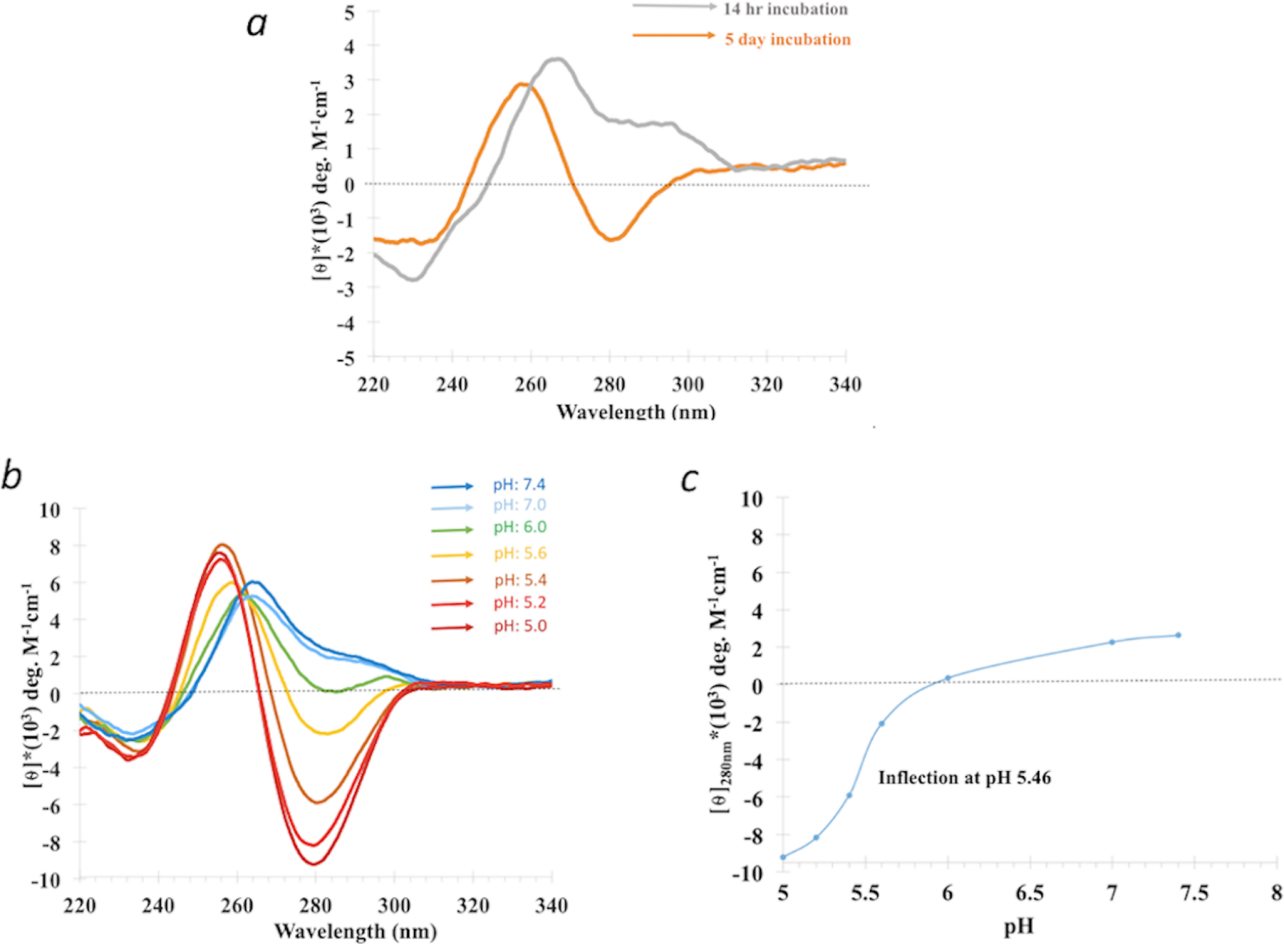
Formation of iCD-DNA. (a) Circular dichroism spectra of 20 μM d(C_2_G_4_)_7_ in TE Buffer plus 150 mM LiCl, pH 7.4. 700 μM DNA in this buffer, at 37°C, was incubated for 14 hrs and for 5 days. CD spectra were taken shortly following dilution to 20 μM DNA, in the same buffer, and measured at 22°C. (b) Circular dichroism spectra of 20 μM d(C_2_G_4_)_7_ in 150 mM lithium citrate buffer at different pH (5, 5.2, 5.4, 5.6, 6) as well as in TE buffer plus 150 mM LiCl (at pH 7.0 and 7.4). 700 μM DNA, in the above buffers, was incubated for 14 hrs at 37°C. CD spectra were taken shortly after dilution to 20 μM DNA, in the appropriate buffer, and measured at 22°C. (c) θ_280_ from Fig 1B plotted as a function of pH.

While preparing a negative control for a CD spectroscopic study of G-quadruplex formation by d(C_2_G_4_)_7_, we observed that this oligomer, dissolved at 700 μM concentration in TE-LiCl buffer (10 mM Tris, pH 7.4, 0.1 mM EDTA, 150 mM LiCl) and incubated at 37° C for up to 5 days, showed an unusual circular dichroism spectrum. Fig 1A shows spectra for 14-hour and 5-day incubations, with the latter being a smooth, inverted CD spectrum (with a maximum at ~255 nm and a minimum, with net negative ellipticity, at ~280 nm). Such a CD spectrum represents an “inversion” of CD spectra typically observed for A-DNA, B-DNA, as well as for classic DNA triplexes and G-quadruplexes [30]. The relative lack of shoulders in the appearance of the inverted spectrum suggested either a unitary DNA species or a series of structurally related species rather than a complexly heterogeneous mixture. The long incubation at relatively high DNA concentration (at least at this pH) that gave rise to this CD signal, also suggested that these were slow-forming, thermodynamically rather than kinetically favored DNA product or products (from this point referred to as “iCD-DNA”).

Given that d(C_2_G_4_)_7_ contains only two of the four nucleobases, G and C, and the known important role of protonated cytosines in the formation of non-canonical secondary DNA structures like triplex and i-motif, we investigated whether pH values of < 7.0 impacted on the inverted CD spectrum. Fig 1B shows that low pH values do indeed accentuate the ellipticity inversion, with amplitudes intensifying even in the 5.2–5.0 pH range and at shorter incubation times than at neutral pH. Fig 1C plots molar ellipticity at 280 nm as a function of pH in the 5.0–7.4 range, obtained from the data in Fig 1B. The dependence was fitted with a sigmoidal function, with an inflection observed at pH 5.46. Roughly, such an inflection pH is consistent with cytosine protonation within the iCD-DNA structure or structures. To check that equilibrium was reached both at high (700 μM) and moderate (20 μM) DNA concentration incubations (analogous to the data shown in Fig 1B), progressively longer incubations under these conditions were carried out. These latter experiments (S1 Fig) also yielded similar computed inflection pH values.

We investigated whether the initial incubation at 700 μM DNA, such as described above, was strictly necessary for the formation of iCD-DNA. S1 Fig shows that d(C_2_G_4_)_7_ incubated at 700 μM in 150 mM lithium citrate, pH 5.2, already shows close to the maximal CD amplitude (observed at 16 hours of incubation) by 30 mins; while, a 20 μM DNA incubation does indeed show the characteristic shape (if not the full CD amplitude) after 16 hours of incubation. These data emphasize that iCD-DNA is a thermodynamically favoured structure that is optimally but not exclusively generated by incubations at relatively high DNA concentrations. However, are long incubations needed at pH 5.2? S2 Fig shows that while 20 μM d(C_2_G_4_)_7_ incubated for 2 hrs at 37° C in buffers of various ionic strengths at pH 7.4, generates species with long-lived low CD ellipticities (corresponding presumably to the unfolded or partially base-paired DNA), incubations in increasing strengths of the Li buffer, all at pH 5.2, yields CD spectra characteristic of iCD-DNA in as little as 2 hrs, as can be seen by comparing with the 14 hr incubation **(**S2 Fig).

**Fig 2:**
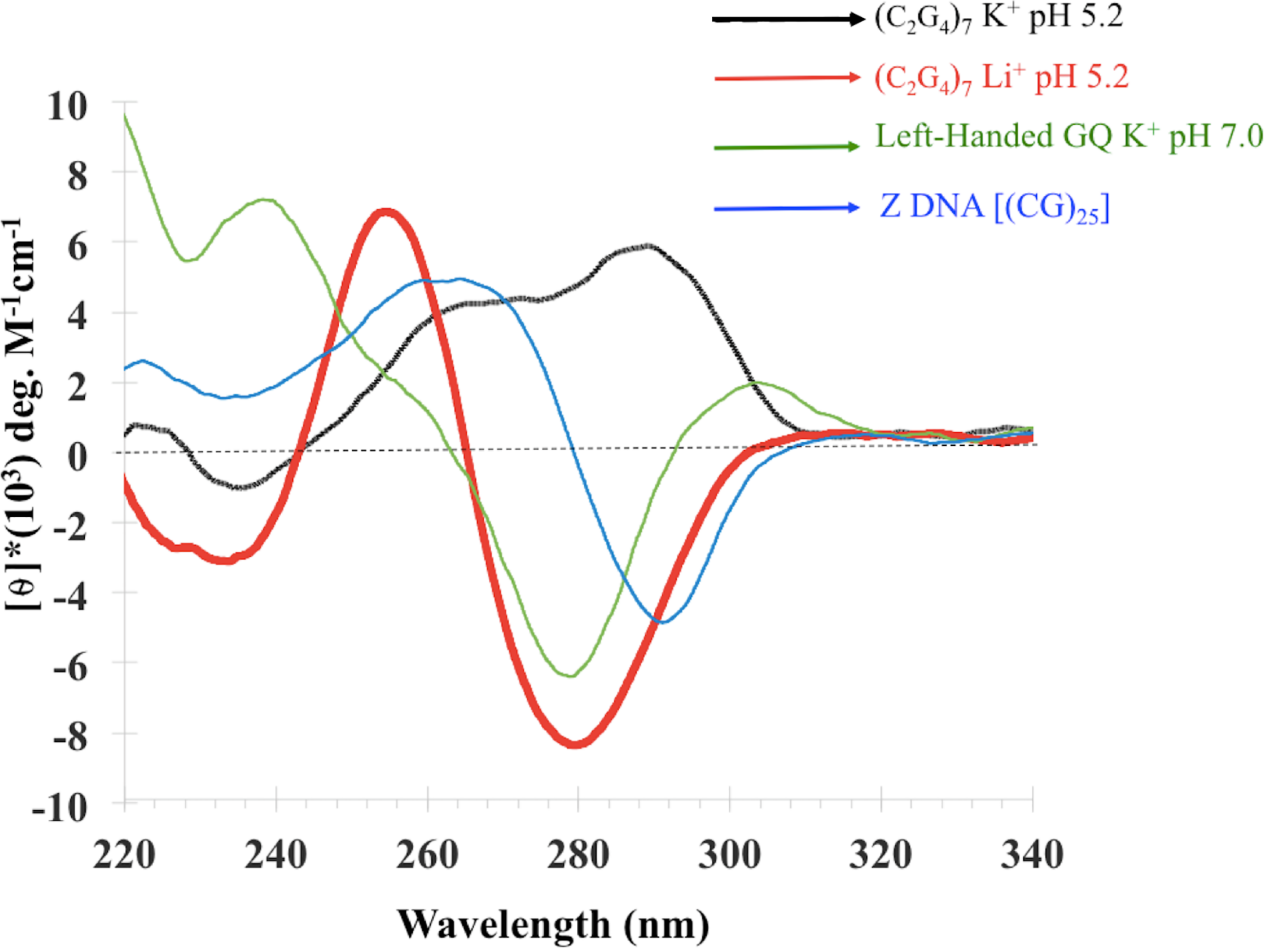
Comparison of iCD-DNA with Z DNA and Z-G4. Circular dichroism spectra of (C_2_G_4_)_7_ in 150 mM potassium citrate, pH 5.2; (C_2_G_4_)_7_ in 150 mM lithium citrate pH 5.2; a left-handed G-quadruplex (GQ) [ZG4: (T(GGT)_4_TG(TGG)_3_TGTT)] in TE (10mM Tris, 0.1mM EDTA, pH 7.0) plus 150 mM KCl; and a duplex Z-DNA [(CG)_25_] in TE plus 4.0 M NaCl.

DNA secondary structures known to show inverted CD spectra include the left handed Z-DNA duplex formed by d(CG)_n_ in 4.0 M Na^+^ [31] as well as one reported instance of a left-handed G-quadruplex (“Z-G4”) formed in the presence of ~100 mM K^+^ at pH 7.0 by the DNA oligomer d[T(GGT)_4_TG(TGG)_3_TGTT] [32]. We measured the CD spectrum of d(CG)_25_ in 4.0 M Na^+^ (pH 7.0), as well as that of the Z-G4 G-quadruplex in 100 mM K^+^ (pH 7.0), and compared them with the spectrum of d(C_2_G_4_)_7_ in 150 mM lithium citrate, pH 5.2. Fig 2 shows these spectra, as well as the spectrum of the K^+^-generated G-quadruplex products formed by d(C_2_G_4_)_7_. It is clear that the “iCD-DNA” spectrum is utterly distinct from that of Z-DNA. With regard to the Z-G4, while its negative molar ellipticity region (270–290 nm) is roughly similar to that of iCD-DNA, the two spectra diverge significantly in the 230–270 region. It is therefore clear that iCD-DNA is not the left-handed Z-DNA duplex, though it may potentially have structural affinities with the one described left-handed G-quadruplex.

### The role of counter-cations in iCD-DNA formation

**Fig 3:**
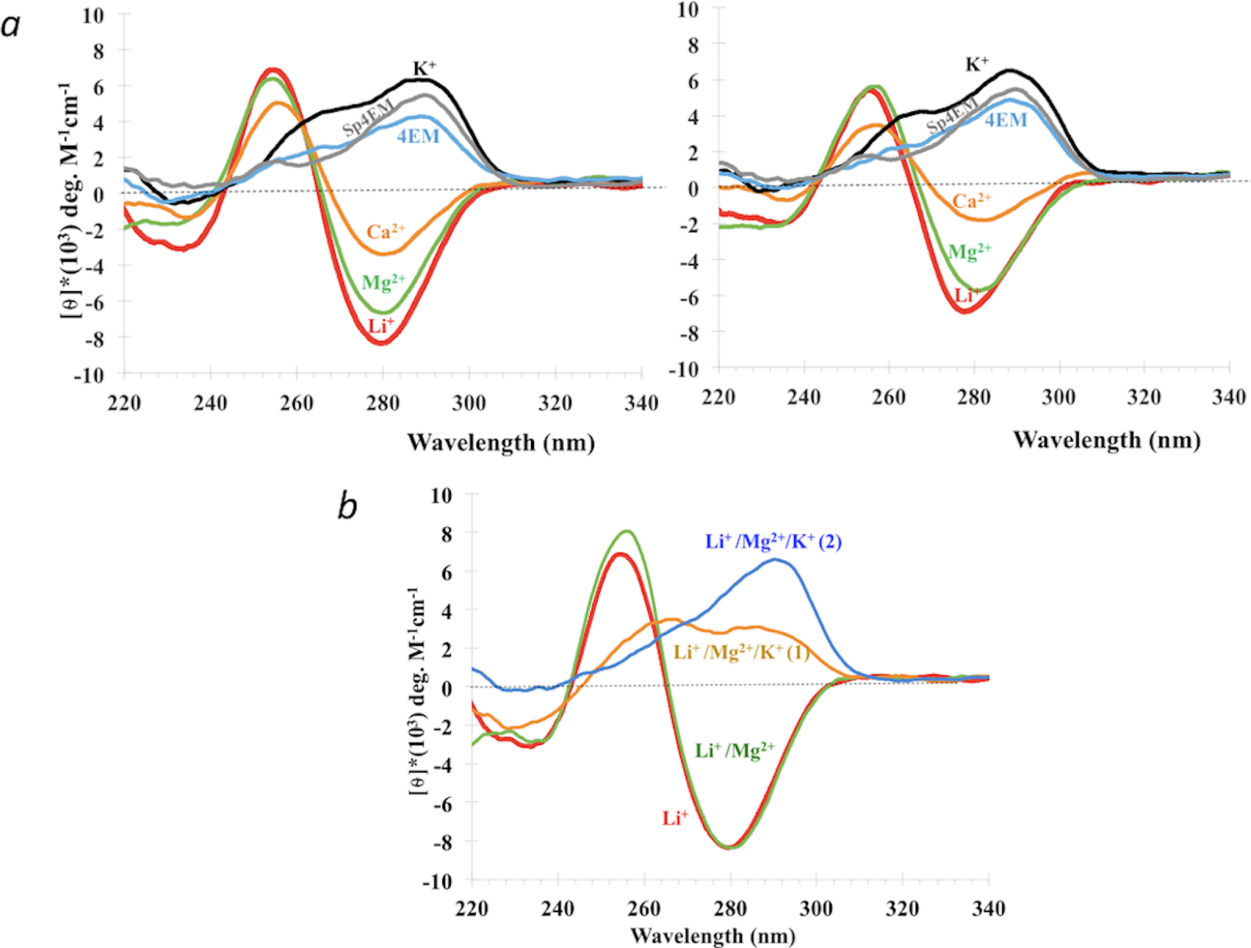
Effect of different counter-cations on iCD-DNA formation and stability. (a) Circular dichroism spectra of 20 μM d(C_2_G_4_)_7_ in various buffered salt solutions at pH 5.2. The spectra on the left were taken immediately following dilution; the spectra on the right were taken 14 hrs following dilution and further incubation in the various buffers at 37° C. (b) CD spectra of 20 μM d(C_2_G_4_)_7_ diluted into different buffers at pH 5.2. All CD measurements were taken at 22°C.

Fig 3A shows the influence, variously, of 150 mM K^+^; 150 mM Li^+^; 10 mM Mg^2+^; 10 mM Ca^2+^; 150 mM 4-ethylmorpholinium^+^ (4EM^+^: pKa = 7.67); and of 50 μM spermine^4+^ plus 150 mM 4EM^+^. To generate the iCD-DNA conformer the following standard protocol was followed: DNA was incubated at 700 μM concentration independently in the above buffers, all at pH 5.2, at 37°C for 14 hrs, following which the solutions were diluted to 20 μM DNA in the same buffers. CD spectra were taken both immediately following dilution as well as after 14 hours of further incubation at 37°C following dilution. Fig 3A, *left* and *right*, show the CD data taken immediately following dilution and 14 hours after dilution, respectively. It can be seen that there is not a large difference in the two sets of spectra. Therefore, once formed, iCD-DNA doesn’t change substantively over time. With regard to the individual incubations, both Li^+^ and Mg^2+^ strongly support iCD-DNA formation; Ca^2+^ does so less efficiently; while the organic cation, 4-ethylmorpholinium, with or without added spermine, does not support it. The K^+^ spectrum refers to G-quadruplex structures formed by d(C_2_G_4_)_7_.

We wished to test for the stability/ persistence iCD-DNA in the presence of K^+^, a cation known specifically to stabilize G-quadruplexes. 700 μM d(C_2_G_4_)_7_ was incubated in 150 mM lithium citrate for 14 hrs at 37°C, followed by dilution to 20 μM of d(C_2_G_4_) in different buffers. Fig 3B shows CD spectra taken in 150 mM lithium citrate, pH 5.2 (“Li^+^”), immediately following dilution; “Li^+^/Mg^2+^”: spectra taken immediately following dilution into 4 mM lithium citrate plus 10 mM magnesium acetate, pH 5.2. “Li^+^/Mg^2+^/K^+^(1)” shows spectra taken 15 mins after dilution into a Li-Mg-K buffer (4 mM lithium citrate, 10 mM magnesium acetate and 25 mM potassium citrate, pH 5.2); and “Li^+^/Mg^2+^/K^+^(2)” shows spectra taken 14 hrs after dilution into the Li-Mg-K buffer. It can be seen that even short incubations at 37° C after addition of K^+^ lead to a disruption of the iCD-DNA spectra, and after 14 hrs in the presence of K^+^, the CD spectra essentially resemble those of G-quadruplex structures formed in K^+^ alone. To determine how much K^+^ could be tolerated in this system, we carried out experiments exactly as above, except with potassium citrate, pH 5.2, added to 10 mM; 1 mM; and 0.1 mM (S3 Fig). The result of the 10 mM K^+^ experiment was similar to those shown in Fig 3B. In 1 mM K^+^, the inverted iCD-DNA spectrum persisted, although with lower amplitude, even after 14 hours of incubation at 37°; in 0.1 mM K^+^, the iCD-DNA spectrum was stable even after 14 hours of incubation.

**Fig 4:**
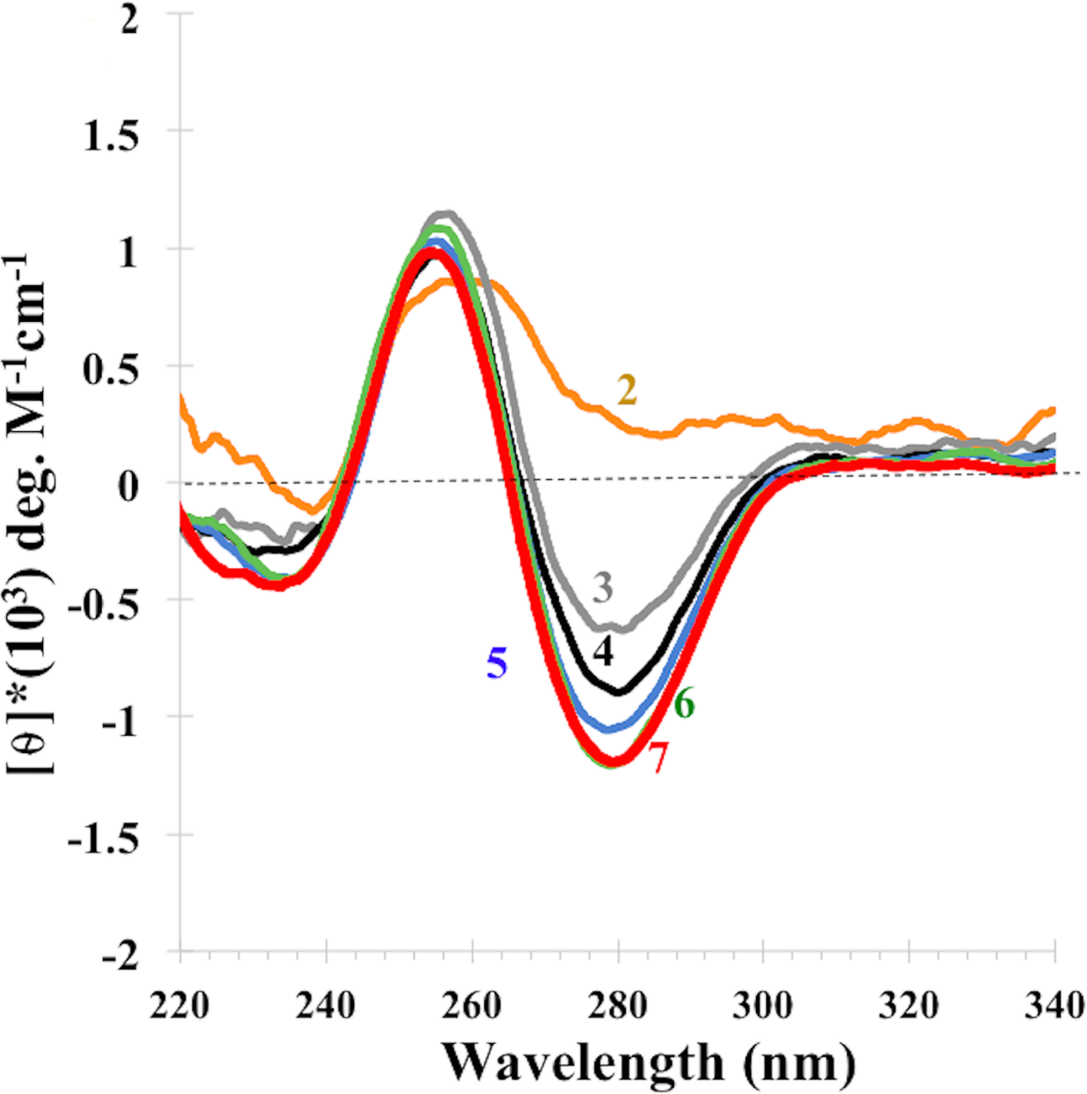
C_2_G_4_ repeat-length dependence on the formation of iCD-DNA. CD spectra of d(C_2_G_4_)_n_ (where n = 2–7), in 150 mM lithium citrate, pH 5.2, at DNA concentrations adjusted to ensure unvarying DNA mass from solution to solution. Each DNA was incubated at 700 μM concentration in 150 mM lithium citrate, pH 5.2, at 37°C for 14 hrs, following which it was diluted to ensure constant DNA mass into the same buffer as follows—“7”: 2.85 μM d(C_2_G_4_)_7_; “6”: 3.33 μM d(C_2_G_4_)_6_; “5”: 4.0 μM d(C_2_G_4_)_5_; “4”: 5.0 μM d(C_2_G_4_)_4_; “3”: 6.66 μM d(C_2_G_4_)_3_; and “2”: 10.0 μM d(C_2_G_4_)_2_.

To investigate whether a minimum number of repeats of (C_2_G_4_) are necessary for iCD-DNA formation, we examined oligomers of the d(C_2_G_4_)_n_ series, where n = 2–7. Fig 4 shows spectra corrected to ensure a constant DNA mass (rather than molar concentration of oligomer), and it can be seen that under these experimental conditions d(C_2_G_4_)_2_ does not form iCD-DNA; the larger oligomers do so progressively, until no further spectral difference can be seen between d(C_2_G_4_)_6_ and d(C_2_G_4_)_7_.

### Do other GC repeats show inverted CD spectra?

**Fig 5:**
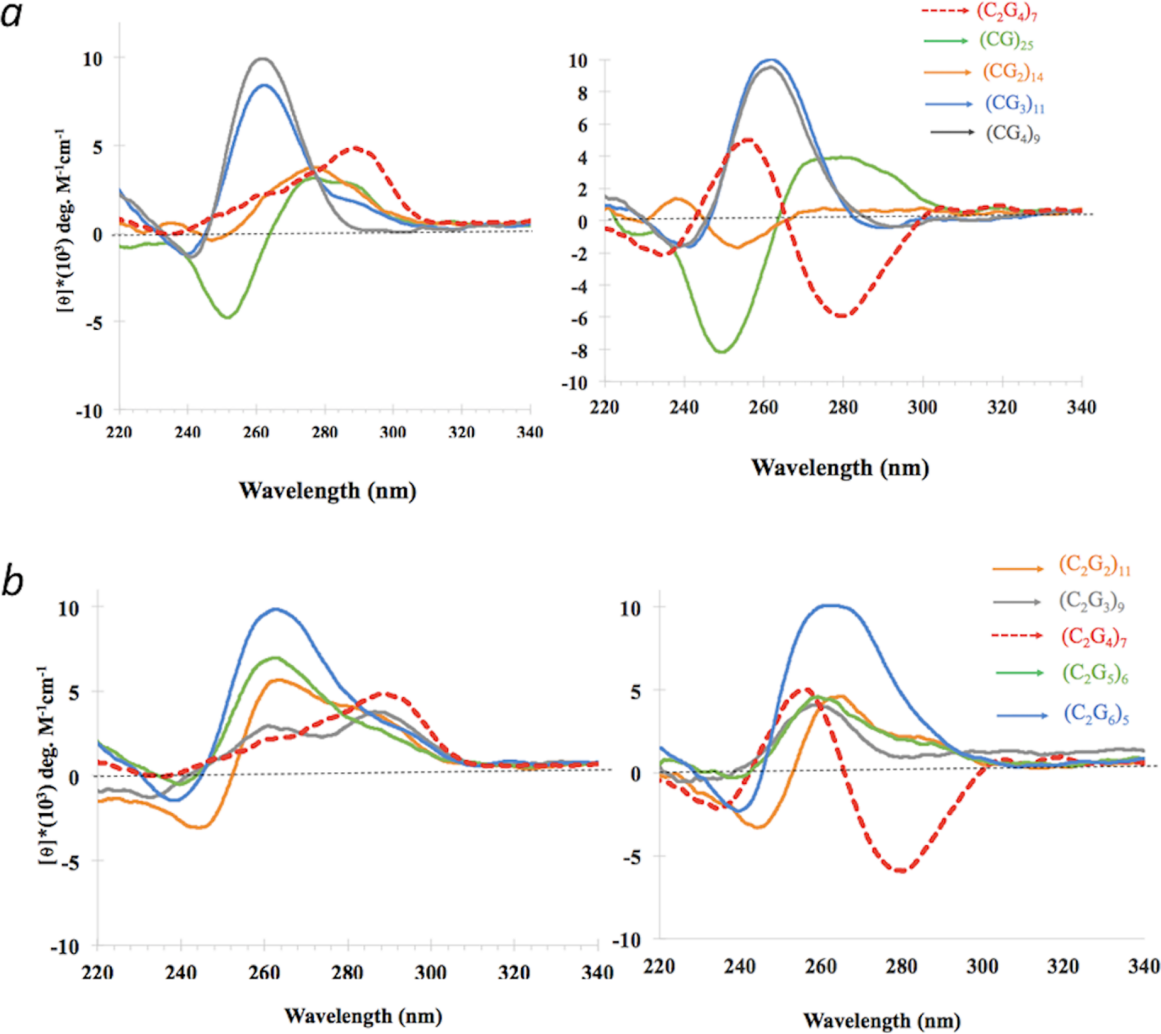
Effect of varying G:C ratios away from C_2_G_4_ on the formation of iCD-DNA. (a-b) Circular dichroism spectra of DNA repeats containing varying C:G ratios, independently incubated and diluted either in 150 mM 4-ethylmorpholine, pH 5.2 (*left*) or 150 mM lithium citrate, pH 5.2 (*right*); in all cases, given the different molecular weights of different oligonucleotides, the DNA mass was kept equal in each solution.

Is the (C_2_G_4_)_n_ sequence unique among GC-rich repeating sequences in forming iCD-DNA? We measured the CD spectra of a number of different GC-rich repeat sequences after the oligomers were incubated in either 150 mM 4EM^+^, pH 5.2 (“4EM buffer”); or in 150 mM lithium citrate, pH 5.2 (“lithium buffer”). Fig 5A and 5B show the CD spectra of a variety of such repeating G/C-rich DNA oligomers. Both figures show that in 4EM buffer (*left*) none of the DNA oligomers shows a spectrum with the inversion features of iCD-DNA; in lithium buffer (*right*) the d(CG_3_)_11_ and d(CG_4_)_9_ oligomers show minor negative molar ellipticities in the 280–300 nm region, though not resembling the iCD-DNA spectrum to any great extent.

**Fig 6:**
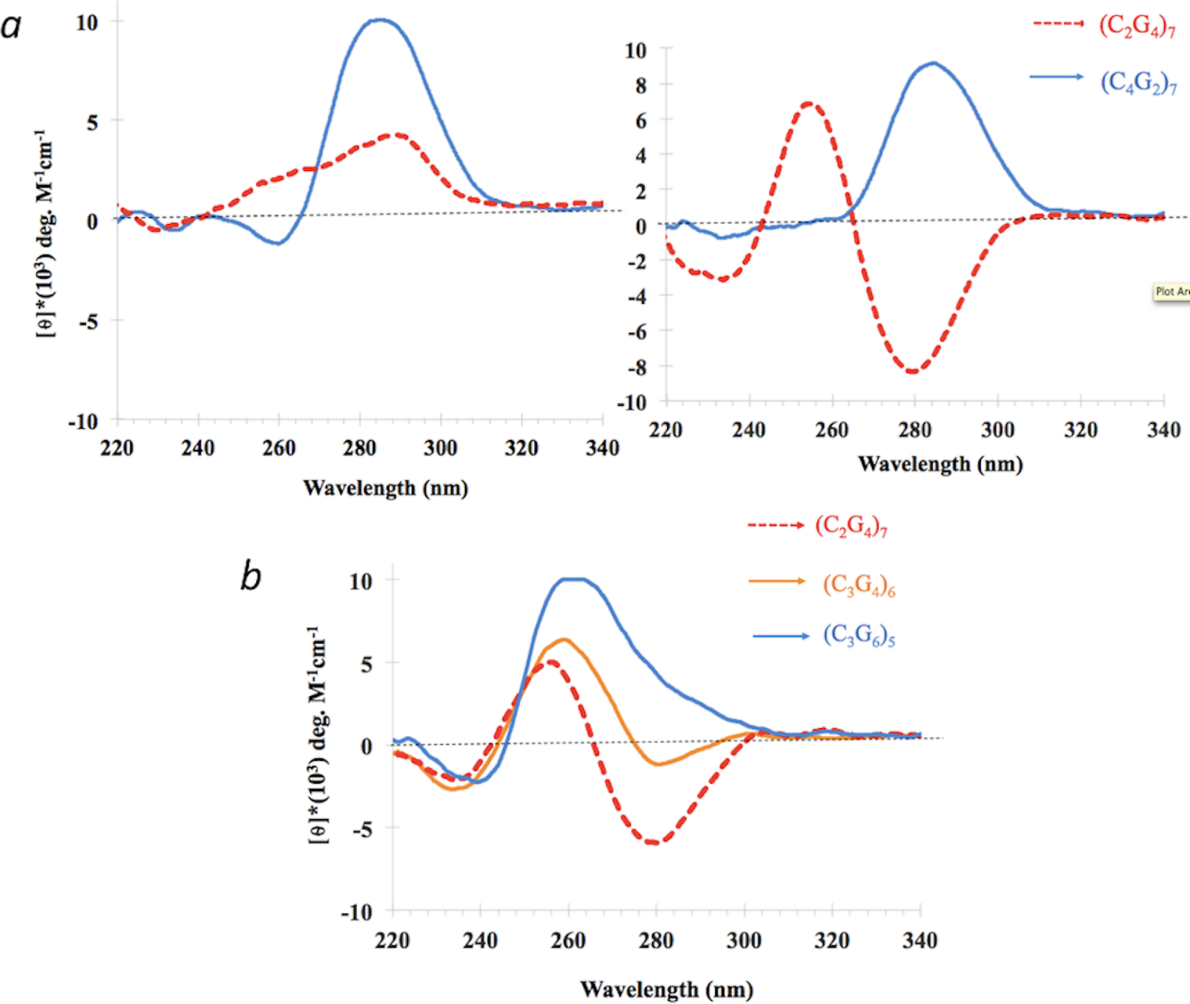
Effect of other G:C ratios on the formation of iCD-DNA. (a) Circular dichroism spectra of DNA repeats containing varying C:G ratios, independently incubated and diluted either in 150 mM 4-ethylmorpholine, pH 5.2 (*left*) or 150 mM lithium citrate, pH 5.2 (*right*); in all cases, given the different molecular weights of different oligonucleotides, the DNA mass was kept equal in each solution; (b) data, as above, but incubated and diluted only in 150 mM lithium citrate, pH 5.2.

Fig 6A compares spectra for d(C_2_G_4_)_7_ with those of its complementary sequence, d(C_4_G_2_)_7_. The d(C_4_G_2_)_7_ sequence, which forms either i-motifs [10] or unusual quadruplexes proposed to contain C-G-C-G quartets [17] in the absence of potassium, does not generate the iCD-DNA spectrum in either incubation solution. Fig 6B shows the spectra of two oligomers, d(C_3_G_4_)_6_ and d(C_3_G_6_)_5_. Like d(CG_3_)_11_ and d(CG_4_)_9_, d(C_3_G_4_)_7_ shows a modest negative molar ellipticity in the 270–300 nm region, but again, its spectrum does not feature the intense negative ellipticity in this region characteristic of d(C_2_G_4_)_7_.

We examined the ability of guanine-rich repeat sequences lacking cytosine to form iCD-DNA. S4 and S5 Figs show that neither d(T_2_G_4_)_7_ nor d(A_2_G_4_)_7_ show the iCD-DNA spectrum over a pH range of 4.0–7.4.

### The melting behavior of iCD-DNA

**Fig 7:**
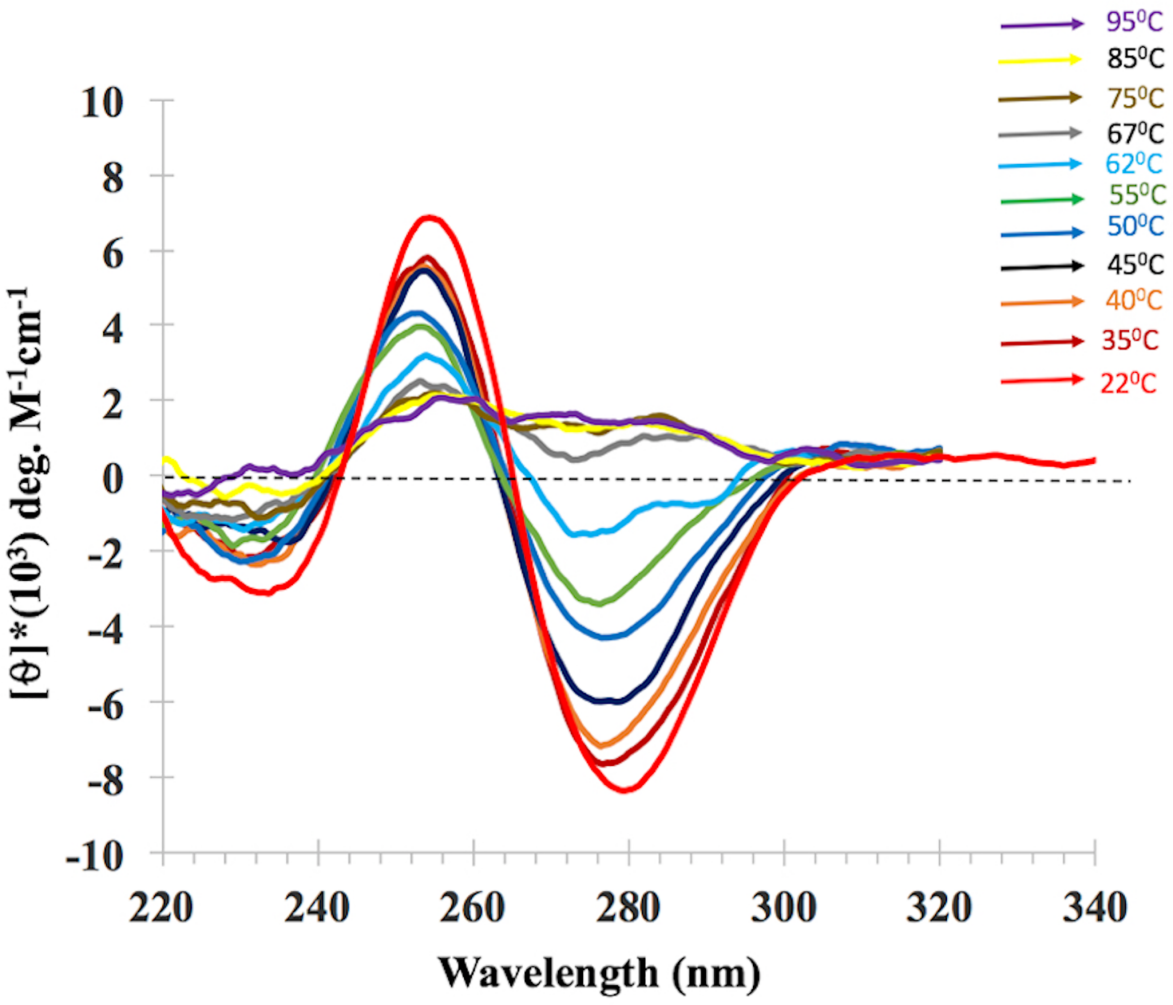
Melting CD spectra of iCD-DNA as a function of temperature. CD spectra of iCD DNA generated from d(C_2_G_4_)_7_, measured as a function of temperature in lithium buffer.

Fig 7 shows the CD spectra of pre-formed iCD-DNA as a function of solution temperature, measured in buffered 150 mM lithium citrate, pH 5.2 (“lithium buffer”). The monotonic decomposition of the inverted CD spectrum (i.e. the lack of appearance of any other classic spectrum corresponding to either A- or B-family DNA duplexes, or canonical triplexes or right-handed G-quadruplexes) indicates that iCD-DNA has a homogenous structure that melts directly to unstructured, single-stranded DNA. S6 Fig plots melting curves obtained by plotting θ_280_ values of d(C_2_G_4_)_7_ in its iCD-DNA form, with data shown both for iCD-DNA in buffered 10 mM magnesium acetate, pH 5.2 (“magnesium buffer”); and in lithium buffer. Smooth two-state melting behaviour is observed in both cases, with T_m_ values calculated at 63°C in magnesium buffer and 60°C in lithium buffer.

### Gel mobility and chemical protection data on iCD-DNA

**Fig 8:**
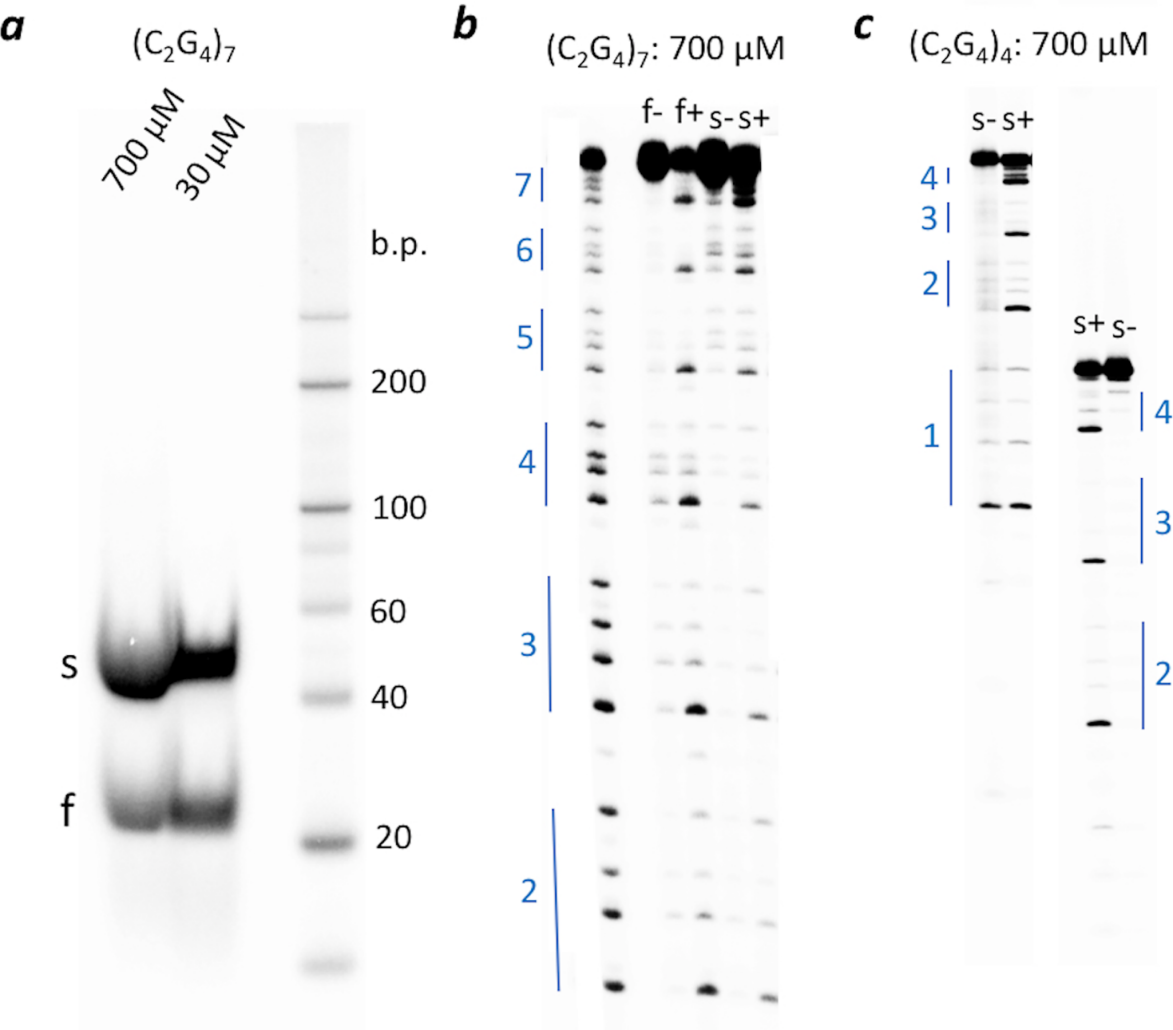
Native gel and DMS footprinting of iCD-DNA. (a) Native gel analysis of iCD-DNA formed from d(C_2_G_4_)_7_; “s” and “f” refer to the slow and fast-moving bands, respectively. (b) DMS-methylation protection data for the “s” and”f” iCD-DNA complexes formed by d(C_2_G_4_)_7_. The lane on the extreme shows a guanine ladder, representing the methylation pattern of unfolded d(C_2_G_4_)_7_. The numbers to the left of the gel indicate the C_2_G_4_ tract number, starting from the 5’ end. “+” as in “f+” refers to DNA samples treated to DMS, followed by hot piperidine; “-”refers to DNA samples treated only with hot piperidine. (c) As in (b), except showing data from iCD-DNA from d(C_2_G_4_)_4_. Two independent loadings are shown to cover all four C_2_G_4_ tracts present in this oligonucleotide.

Whether iCD-DNA consists of a single or multiple molecular species was examined by native gel electrophoresis. d(C_2_G_4_)_7_ was first incubated, at different DNA concentrations (30 and 700 μM), for 1 or 14 hours at 37°C in lithium buffer. The resulting incubations were run in a 7.5% polyacrylamide non-denaturing gel run in TAE-Li buffer, pH 5.2. Fig 8A shows the data. Curiously, both sets of incubations gave rise to two distinct electrophoretic bands (“s”: slower, and “f”: faster). The same result was found with a 700 μM incubation of d(C_2_G_4_)_4_, though the “s” band was overwhelmingly abundant for this oligomer. How robust were these “f” and “s” complexes—did their relative distribution in the native gel reflect their relative abundance in solution? To test this, “f” and “s” complexes from the lithium incubations of d(C_2_G_4_)_7_ were excised and eluted from the native gel into lithium buffer, concentrated to ~5 μM without resorting to ethanol precipitation, and re-run into the native gel (TAE-Li buffer, pH 5.2). S7 Fig shows that ≥ 90% of each purified complex re-ran with its characteristic gel mobility. This suggests that the two complexes are generally stable and not in a rapid dynamic equilibrium under our incubation and dilution conditions.

Dimethyl sulfate (DMS) was used to try and define the base-pairing within the “s” and “f” complexes from the lithium buffer incubations. DMS selectively methylates guanines at their N7 position, which can be involved in Hoogsteen/Reverse Hoogsteen but not in Watson-Crick base pairing. Fig 8B shows a 20% denaturing gel with the protection data for “f” and “s” complexes formed by d(C_2_G_4_)_7_, and Fig 8C shows the data for the predominant “s” band formed by d(C_2_G_4_)_4_. A striking observation is that in all cases, the same distinctive methylation pattern can be seen, in which only the 5’-most guanine in a given GGGG stretch reacts strongly with DMS, while the other three are only modestly reactive or unreactive. Since DMS-methylation was carried out in the 30 μM or 700 μM DNA solution prior to loading on the native gel, it is therefore reasonable to deduce, since interconversion of the “f” and “s” complexes does not appear to be facile (S7 Fig), that the “f” and “s” products represent fundamentally the same iCD-DNA structure, varying only in their strand stoichiometries.

**Fig 9:**
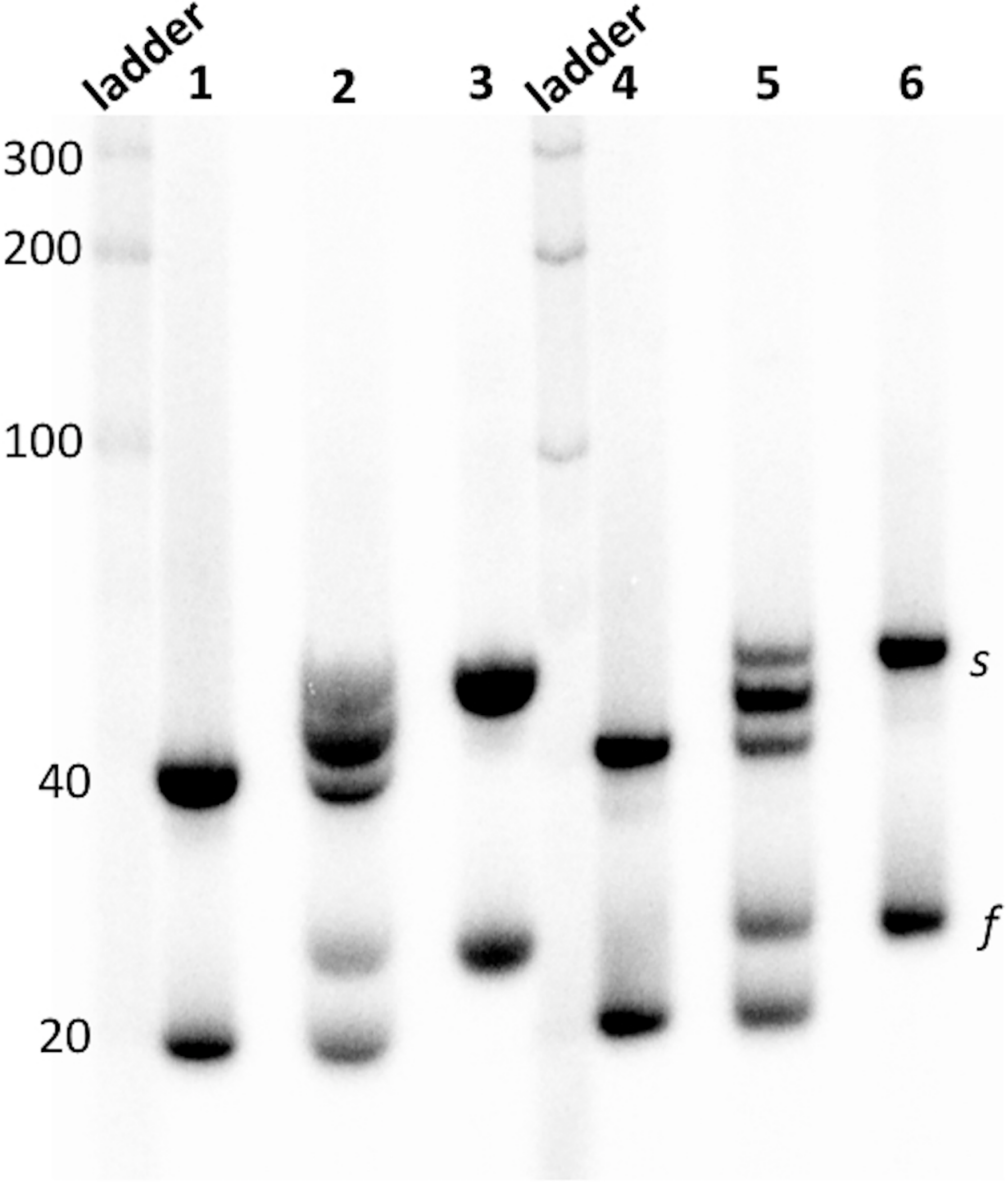
Investigation of the strand stoichiometries of the “f” and “s” products. Lane 1–3 show incubations with [DNA] = 700 μM and lanes 4–6 show incubations with [DNA] = 30 μM. Lanes 1 and 4 show incubations of d(C_2_G_4_)_7_; lanes 3 and 6 show incubations of d(C_2_G_4_)_7_T_6_; and, lanes 2 and 5 show incubations of equimolar mixes of d(C_2_G_4_)_7_ and d(C_2_G_4_)_7_T_6_. The numbers shown to the left of the double-stranded ladder show the values of individual bands as base pairs.

Fig 9 shows a mixing experiment designed to investigate the strand stoichiometries of the “f” and “s” products seen in Fig 8A. A slightly larger oligonucleotide than (C_2_G_4_)_7_ was synthesized by adding a T_6_ stretch to the 3’ end, to give a (C_2_G_4_)_7_T_6_ oligonucleotide. (C_2_G_4_)_7_ and (C_2_G_4_)_7_T_6_, were now allowed to form iCD-DNA either individually (lanes 1, 3, 4, and 6), or as a mixture [equimolar (C_2_G_4_)_7_ and (C_2_G_4_)_7_T_6_] (lanes 2 and 5). Fig 9 shows that from the mixtures, two distinct “f” bands formed while three distinct “s” bands formed (lanes 2 and 5). This is consistent with the “s” complex being a strand dimer and the “f” complex being a strand monomer (thus, the three “s” products seen from the mixture corresponding to [(C_2_G_4_)_7_]_2_; (C_2_G_4_)_7_•(C_2_G_4_)_7_T_6_; and [(C_2_G_4_)_7_T_6_]_2_).

To test whether the distinctive methylation pattern seen for (C_2_G_4_)_7_ incubated in pH 5.2 lithium buffer (only the 5’-most G out of a GGGG stretch reacting strongly with DMS) is uniquely associated with iCD-DNA, we carried out DMS-methylation experiments on (C_2_G_4_)_7_ incubations in pH 5.2 magnesium buffer (which also supports iCD-DNA formation, as defined by CD spectroscopy) and in pH 5.2 4EM and pH 5.2 potassium buffers (neither of which supports iCD-DNA formation). S8 Fig shows that the methylation data in pH 5.2 magnesium buffer closely resembles the pattern found in pH 5.2 lithium buffer. S9 Fig, however, shows that in pH 5.2 4EM buffer, neither the “f” nor the “s” complexes show methylation patterns characteristic of iCD-DNA (Fig 8B and 8C and S8 Fig). Notably, *both* the 5’-most and 3’-most guanines of a given GGGG tract are reactive to DMS. In pH 5.2 potassium buffer, expected to form G-quadruplexes, the methylation patterns are generally faint but resemble the pH 5.2 4EM buffer patterns more closely than those obtained from the two iCD-DNA supporting buffers. Most interestingly, pH 7.0 lithium buffer (S10 Fig) gives methylation patterns for “f” and “s” that are distinct from each other, and both are very distinct from the iCD-DNA methylation signature. The “f” pattern resembles the G-ladder; whereas, the “s” pattern closely the pH 5.2 4EM pattern (S9 Fig). It is clear therefore that both Li^+^/Mg^2+^ *and* low pH are required for the distinctive methylation pattern (as well as CD signature) of iCD-DNA.

We investigated the methylation pattern of two other G/C-rich repeat sequences, d(CG_4_)_9_ and d(CG_3_)_11_, which have roughly the same molecular weight as d(C_2_G_4_)_7_. Neither of these two new repeats shows the inverted CD signature characteristic of iCD-DNA (see Fig 5A). S11 Fig shows that in native gels run at pH 5.2, 700 μM oligonucleotide concentrations of d(CG_4_)_9_ and d(C_2_G_4_)_7_ both run as two bands each, fast (f) and slow (s). Methylation data of these various products are also shown in S11 Fig. It can be seen that the d(CG_4_)_9_-s and d(CG_4_)_9_-f complexes show methylation patterns distinct from each other as well as from d(C_2_G_4_)_7_-s and d(C_2_G_4_)_7_-f [the two d(C_2_G_4_)_7_ complexes, of course, show similar patterns, with the 5’-most guanine in any **G**GGG stretch strongly methylated and the remaining three poorly/not methylated]. Strikingly, the (CG_4_)_9_-s complex shows the *second* guanine of each of its G**G**GG strongly methylated.

S12 Fig shows the analogous native gel and methylation patterns for the “f” band formed from (CG_3_)_11_. Again, this methylation pattern is utterly different from those of the (C_2_G_4_)_7_-s and (C_2_G_4_)_7_-f complexes.

That distinctive **G**GGG iCD-DNA methylation pattern of iCD-DNA formed by d(C_2_G_4_)_7_, however, does not immediately suggest a specific higher-order structure; most likely, there are a number of possible higher order folds of DNA can give rise to this methylation pattern. A methylation pattern alone is often insufficient to predict a detailed structure, given uncertainties about what kind of base-pairing may or may not occur particularly in various G-G base pairings. Nevertheless, this DMS protection pattern is useful to take into account for the building of one or more structural models for iCD-DNA, which are discussed, below.

### Structural models for iCD-DNA

To list what the above experiments reveal about iCD-DNA, we have the following: (a) an acidic pH is required for iCD-DNA formation; suggesting that the protonation of one or both cytosines in each C_2_G_4_ repeat is likely an important contributor; (b) the DMS methylation data show distinctive and consistent pattern, with the 5’-most G of each GGGG stretch reactive to DMS, and the others substantially protected; this holds true for both the “f” and “s” bands of iCD-DNA seen in acidic native gel (suggesting that “f” and “s” are effectively the same complex albeit with different strand molecularity; (c) Li^+^ and Mg^2+^ cations are required for iCD-DNA formation; Ca^2+^ is only marginally effective, and a bulky organic monovalent cation, 4EM^+^, is ineffective. Spermine^4+^ is also ineffective. (d) The inverted CD signature of iCD-DNA suggests it is a structure not yet recorded in the literature [30]; the partial similarity of this CD spectrum to that of one reported instance of a left-handed G-quadruplex [32] indicate that iCD-DNA may be an unusual variant of the classic G-quadruplex (which normally requires Na^+^, K^+^, or Sr^2+^ cations to form); indeed, we find that iCD-DNA converts relatively efficiently to classic G-quadruplexes when K^+^ is added to iCD-DNA in lithium buffer. (e) The two-state melting curve suggests the formation of a homogenous structure, which directly melts to unstructured single stranded DNA. Certain classes of DNA helical structures, such as triple helices, generally show more complex melting behavior, with the Hoogsteen/Reverse-Hoogsteen bonded third strand melting away from the duplex at the lower temperature than the duplex itself, though there have been reports of the two-melting transition (i.e. between three states) located close to each other [33–39]. Thus, the observation of a single melting event between two states for iCD-DNA is not in itself sufficient to rule out the possibility of a conventional triplex, although the strand composition of d(C_2_G_4_)_7_ is not formally suitable for forming a canonical YRR or YRY triplex.

It is possible to eliminate certain classes of higher-order DNA structure for iCD-DNA. First, the uniquely inverted CD spectrum of iCD-DNA rules out the possibility of B- or A- family double helices [30]; right-handed Hoogsteen duplexes [40], as well as conventional, right-handed G-quadruplexes [9] and classic i-motif structures [10].

**Fig 10:**
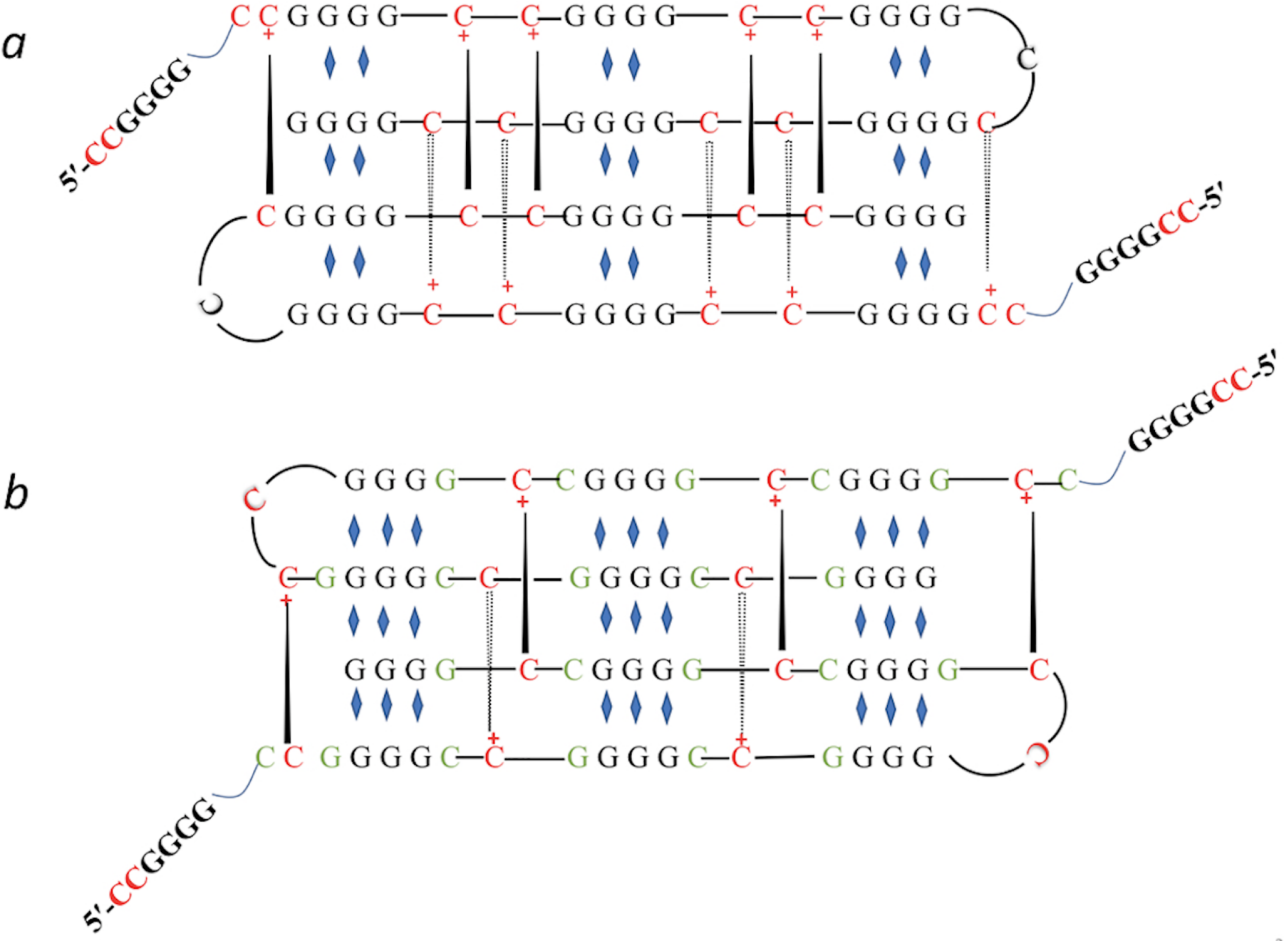
Left-handed hybrid i-motif/G-quadruplex model for iCD-DNA. A structural model for iCD-DNA based on i-motif and G-quadruplex elements. (a) A model consisting purely of alternating i-motif and G-quadruplex motifs. (b) A related model, but one that contains GCGC quartets in addition to the i-motif and G-quadruplex. In the above diagrams, the blue diamonds represent Hoogsteen hydrogen-bonding interactions, while the black lines indicate C-C^+^ bonding such as found in i-motifs.

So, what could iCD-DNA’s structure be? Protonated cytosines are known to participate in Hoogsteen/Reverse Hoogsteen bonding [40, 41] as well as in forming i-motifs [42]. Most simply, iCD-DNA could be left-handed Hoogsteen-bonded duplexes, “f” being an intramolecular folded form, and “s” an intermolecular form involving two distinct strands. However, two further classes of structure we propose here (below) do involve protonated cytosines in more complex structures. While d(C_2_G_4_)_n_ repeats contain bases that normally Watson-Crick base-pairs with each other, the requirement for acid pH to form iCD-DNA suggest that i-motifs may still be forming, even given the 2:1 excess of guanines over cytosine in the d(C_2_G_4_)_n_ repeats. We propose that iCD-DNA may consist of short i-motifs stretches separating loose (i.e. not stabilized by K^+^) G-quadruplexes, which could well be left-handed and so contribute to the inverted CD spectrum of iCD-DNA (Fig 10). Two alternative structures can be contemplated, which differ in the specifics of base-pairing. Fig 10A shows a structure that contains only the i-motifs and loose G-quadruplexes, the interdigitated structure of i-motifs helping to hold together the Li^+^ (or Mg^2+^ but not 4EM^+^)-stabilized, relatively loose G-quadruplex, whose outermost guanines (typically, only the 5’ G of a given run) could be susceptible to DMS-methylation. Fig 10B shows a possible variant of the above structure, this one incorporating GCGC base quartets in addition G-quartets. Classic GCGC quartets have been observed in high-resolution structures of certain G-quadruplexes [43]. The “f” and “s” bands seen in the native gel of iCD-DNA refer to monomeric and dimeric complexes, respectively (*vide infra*).

**Fig 11:**
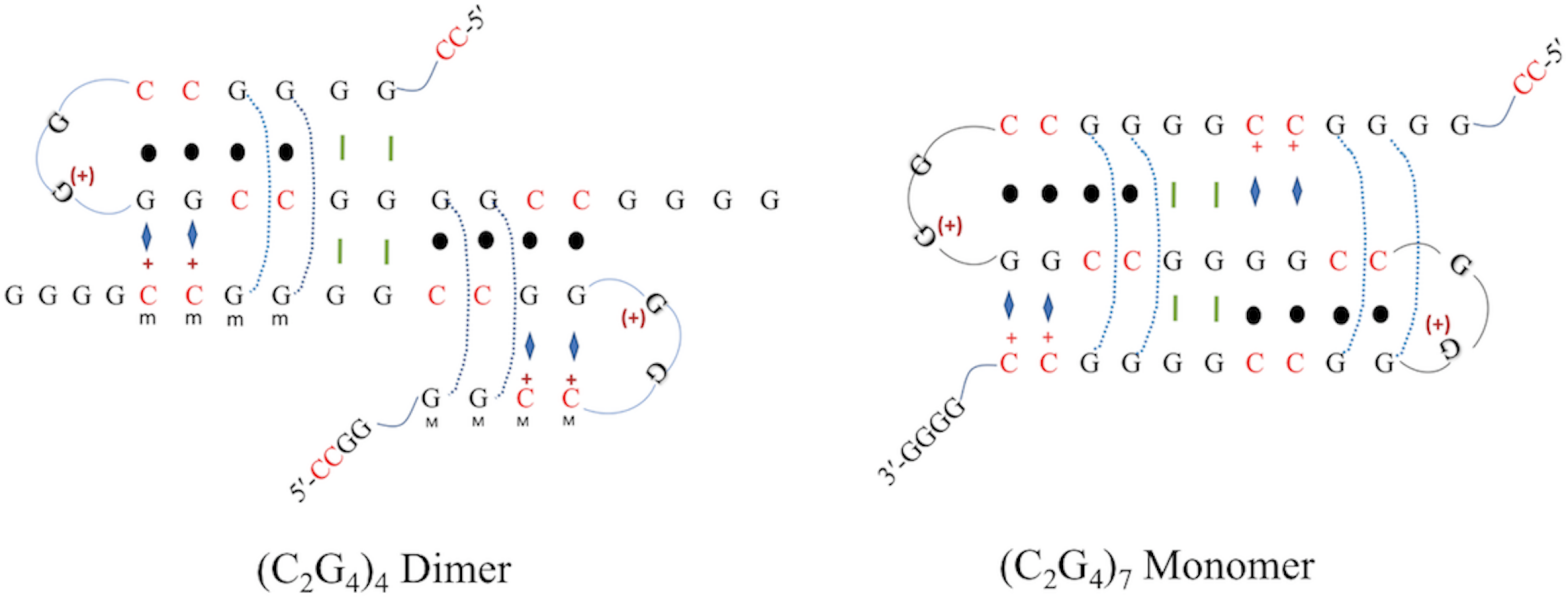
A braided triplex model for iCD-DNA. Braided, Watson-Crick base-pairing founded structural model. Black dots indicate Watson-Crick base pairing; blue rhomboids as well as the blue dotted lines indicate Hoogsteen/Reverse Hoogsteen interaction. Green bars indicate hydrogen-bonding within G-triple buffer zones. The Watson Crick interactions involve switching of base-pairing partners involving a specified strand and the other two available strands, facilitated by the buffer zone of two consecutive ‘G-triples’ in both the braided model (C_2_G_4_)_4_ Dimer and (C_2_G_4_)_7_ Monomer.

Alternatively, given the highly symmetric nature of the repetitive sequence (C_2_G_4_)_n_, the potential exists for the formation of a non-canonical, braided or entangled structures, founded on Watson crick base-pairing between guanines and cytosines. Braiding occurs via ‘partner swapping’ of strands (or stretches of a given strand) participating in Watson-Crick base-pairing (Fig 11 shows two versions of such a ‘braided’ complex). The alternation of strands participating in Watson-crick base-pairing could be facilitated by conformationally fluid “buffer zones” made up of two consecutive G-triples. Such braided structures have been proposed by Bai and Colleagues to form from λ phage DNA; these authors carried out a computational simulation that featured alternating left-handed and right-handed helical elements [44, 45]. Superficially, such braided structures would resemble DNA triplexes; although canonical triplexes (YRY and YRR, stabilized by Mg^2+^, polyamines, and/or low pH) typically do not show inverted CD spectra, modest inversion (or close to zero ellipticity) has been observed at ~280 nm from certain “anti-parallel” triplexes where the third strand has a mixed purine and pyrimidine content [33]. Of course, the d(C_2_G_4_)_n_ sequence is unsuitable for forming canonical YRY and YRR triple helices (in which very little deviation is tolerated to the strict requirement for one all-purine and one all-pyrimidine strand forming a Watson-Crick duplex to which a third strand (all purine / all pyrimidine /purine-pyrimidine mixture) binds [46].

One prediction about such braided structures is that topological entanglement of the strands should override the strict canonical rules that hold for conventional triplexes (such as the requirement for the third strand to be anti-parallel to the duplex’s purine strand in YRR triplexes and parallel to that strand in YRY triplexes). Each entrapped GGCC “third strand” stretch in a braided complex would therefore base-pair either conventionally (i.e. via Hoogsteen or reverse Hoogsteen base pairing) or unconventionally with the Watson-Crick base-paired tract adjacent to it. Another prediction is that in order to remain conventionally right-handed, the ‘third strand’ would need necessarily to alternate between lying in the major and minor grooves of the duplex. Precedent for minor-groove-bound third strands exist in RNA triplexes [47]. Alternatively, if the third-strand disposition within each triplex tract of iCD-DNA were required to be uniformly in the duplex’s major groove, the triplex tracts would need to alternate from being left-handed and right-handed helices. Such dramatic changes in helical direction from tract to tract could, again, be enabled by conformationally fluid G-triple “buffer zones”. In such braided structures, since non-canonical base triples would be expected to form, they could confound our ability to interpret the DMS methylation that we report here.

## Conclusion

We have reported here an unusual DNA structure—iCD-DNA—characterized by an inverted circular dichroism (CD) spectrum in the 220–310 nm wavelength range. iCD-DNA formation shows a pH dependence and optimizes in the pH range of 5.0–5.2. The inflection pH at equilibrium, for high DNA concentration (700 μM) incubations, is ~5.85, consistent with the pK_a_ for cytosine protonation (given that the inflection pH, even at equilibrium, may not precisely equate a pK_a_ value [48, 49]). With our data, we are not able yet to propose a definitive structural model for iCD-DNA. Under our experimental conditions, native gel analysis shown that two distinct species, albeit most likely of similar or identical structure, are obtained. This militates against immediate high-resolution structure determination using NMR spectroscopy or X-Ray crystallography. We have therefore proposed three general categories of structure that would likely be consistent with all the experimental data that we have obtained. The repeating (C_2_G_4_)_n_ DNA sequence from the human *C9orf72* gene is causally linked to the development of a number of neurodegenerative diseases (*vide infra*). This particular, very guanine-rich, repeat is known to favor the formation of G-quadruplexes in the presence of the potassium ion, the dominant monovalent cation in the intracellular environment. By contrast, the iCD-DNA structure reported here is destabilized by the presence of potassium ions. However, iCD-DNA formation is promoted by the Mg^2+^ ion, which is present in millimolar concentrations in the cell; so, the physiological relevance of iCD-DNA under specialized intracellular conditions cannot be ruled out. It is known that significant fluctuations of potassium ion can occur in the cell [50, 51]. However, in considering a broader picture of repeat expansion DNA sequences [52, 53], many of which do not form G-quadruplexes but do form foldback hairpin structures, it is conceivable that with sufficiently long repeats, Watson-Crick base-paired tracts may switch strands, as in iCD-DNA, to give rise to braided structures; or, indeed, form non-conventional left-handed i-motif / G-quadruplex hybrids. Proposals somewhat akin to this have been made recently [54].

## Supporting information

S1 Fig Kinetics and pH dependence of iCD-DNA formation in acidic condition. CD spectra of d(C_2_G_4_)_7_ incubated at 700 μM (*left*) and 20 μM (*right*) in 150 mM lithium citrate, pH 5.2. Upper left: 700 μM d(C_2_G_4_)_7_ incubated in 150 mM lithium citrate, pH 5.2, at 37° C, for the time indicated, followed by dilution to 20 μM of d(C_2_G_4_)_7_ in 150 mM lithium citrate and immediate CD measurement. Upper right: 20 μM d(C_2_G_4_)_7_ incubated in 150 mM lithium citrate, pH 5.2, at 37° C for the times indicated. Incubations carried out under the two conditions, above, for 3 days, gave superimposable CD spectra. Middle left: CD spectra of 700 μM d(C_2_G_4_)_7_ incubated in 150 mM lithium citrate, at different pH values, for 5 days at 37° C, then diluted to 20 μM DNA in the buffer of the same pH. Following dilution, the CD spectra were measured immediately. Middle right: θ_280_ values from figure at middle left, plotted as a function of pH. Lower left: CD spectra of 700 μM d(C_2_G_4_)_7_ incubated in 150 mM lithium citrate, at different pH values, for 5 days at 37° C, then diluted to 20 μM DNA in the buffer of the same pH, followed by incubation at 37° C for a further 14 hours prior to CD measurement. Lower right: θ_280_ values from figure at lower left, plotted as a function of pH.
(TIFF)

S2 Fig iCD-DNA formation as a function of pH and Li^+^ concentration. (a) CD spectra of 20 μM d(C_2_G_4_)_7_, incubated for 2 hrs at 37° C in buffers of various ionic strengths, all at pH 7.4. (b) and (c) CD spectra of 20 μM d(C_2_G_4_)_7_, incubated at 37° C in different concentrations of Li buffer, pH 5.2, for 2 hrs (b); and for 14 hrs (c).
(TIFF)

S3 Fig Effect of potassium on the persistence of iCD-DNA. CD spectra of 20 μM d(C_2_G_4_)_7_ diluted into different buffers at pH 5.2. All CD measurements were taken at 22°C.
(TIFF)

S4 Fig CD spectra of (T_2_G_4_)_7_ as function of pH at 150 mM lithium citrate. Circular dichroism spectra of 20 μM d(T_2_G_4_)_7_ in 150 mM lithium citrate buffer at different pH values (4.0–6.0); as well as in TE buffer plus 150 mM LiCl (pH 7.0 and 7.4). 700 μM DNA, in the above buffers, was incubated for 14 hrs at at 37°C. The CD spectra, taken at 22°C, were taken shortly following dilution to 20 μM DNA in the same buffers.
(TIFF)

S5 Fig CD spectra of (A_2_G_4_)_7_ as function of pH at 150 mM lithium citrate. Circular dichroism spectra of 20 μM d(A_2_G_4_)_7_ in 150 mM lithium citrate buffer at different pH values (4.0–6.0) and in TE buffer plus 150 mM LiCl (pH 7.0 and 7.4). 700 μM DNA, in the above buffers, was incubated for 14 hrs at 37°C. The CD spectra, measured at 22°C, were taken shortly following dilution to 20 μM DNA in the same buffers.
(TIFF)

S6 Fig Melting curves of iCD-DNA in Li^+^ and Mg^2+^. Melting curves (molar ellipticity at 280 nm as a function of temperature) for iCD-DNA generated from incubation of d(C_2_G_4_)_7_ in 10 mM magnesium acetate, pH 5.2; and, in 150 mM lithium citrate, pH 5.2. Rate of heating was 5° C/min.
(TIFF)

S7 Fig Native gel to check the interconversion of ‘s’ and ‘f’ species of iCD-DNA. Re-run “f” and “s” species show a lack of interconversion. ‘s’ and ‘f’ species were excised and eluted from an initial native gel (Fig 8A), concentrated, then re-run on a native gel. 1: species ‘f’ from the 30 μM DNA incubation, Li^+^ lane. 2: species ‘s’ from the 30 μM DNA incubation, Li^+^ lane. 3: species ‘f’ from the 700 μM DNA, Li^+^ lane. 4: species ‘s’ from the 700 μM DNA incubation, Li^+^ lane. The duplex ladder on the far right has its band sizes indicated in base pairs.
(TIFF)

S8 Fig DMS footprinting of iCD-DNA in Mg^2+^. Methylation patterns of “f” and “s” bands obtained from incubation of 100 μM d(C_2_G_4_)_7_ in magnesium buffer, pH 5.2, at 37°C for 14 hours. DMS-methylation was performed on the DNA prior to separation of “f” and “s” bands in a native gel run in TAE buffer, pH 5.2. The purified DNA was treated at 90°C with 10% v/v piperidine prior to analysis on the above denaturing gel. The bands on the left and right side of the gel represent loadings at different times on the gel, to enable visualization of all seven repeats of (C_2_G_4_) in the d(C_2_G_4_)_7_ oligonucleotide forming the iCD-DNA.
(TIFF)

S9 Fig DMS footprinting of (C_2_G_4_)_7_ in 4EM^+^, Li^+^ (iCD-DNA) and K^+^ at pH 5.2. (a) native gel run in TAE buffer, pH 5.2, showing the products of incubation (at 30 μM and 700 μM DNA) of d(C_2_G_4_)_7_ in Li buffer, K buffer, and 4 EM buffer, all at pH 5.2. DMS-methylation was performed on the DNA incubations prior to separation of “f” and “s” bands in the native gel. (b) The purified DNA was treated at 90°C with 10% v/v piperidine prior to analysis on the denaturing gel. The bands shown correspond to the 700 μM (C_2_G_4_)_7_ incubations. “-”and “+” refer to the absence or presence of DMS treatment. The red dots in the lithium buffer data indicate the strongly methylated 5’-most G out of each GGGG stretch in the f+ and s+ lanes.
(TIFF)

S10 Fig DMS footprinting of (C_2_G_4_)_7_ in lithium buffer at pH 7. Methylation patterns of “f” and “s” bands obtained from incubation of 700 μM d(C_2_G_4_)_7_ in lithium buffer, pH 7.0, at 37°C for 14 hours. DMS-methylation was performed on the DNA prior to separation of “f” and “s” bands in a native gel run in TBE buffer, pH 8.0. The purified DNA was treated at 90°C with 10% v/v piperidine prior to analysis on the above denaturing gel.
(TIFF)

S11 Fig DMS footprinting of (CG_4_)_9_ as compared to (C_2_G_4_)_7_ in 150 mM lithium citrate, pH 5.2. (a) Native gel, run in TAE buffer, pH 5.2, showing “f” and “s” bands formed from 30 μM of d(C_2_G_4_)_7_ and of d(CG_4_)_9_ in Li buffer, pH 5.2. (b) Denaturing gel showing methylation data from the above. Lanes 1, 7: G-ladder, no DMS. Lanes 2, 8: G-ladder, yes DMS. Lanes 3, 9: “f” bands, no DMS. Lanes 4, 10: “f bands, yes DMS. Lanes 5, 11: “s” bands, no DMS. Lanes 6, 12: “s” bands, yes DMS.
(TIFF)

S12 Fig Native Gel and DMS methylation analysis for (CG_3_)_11_ in 150 mM lithium citrate, pH 5.2. (a) Native gel, run in TAE buffer, pH 5.2, showing the “f” and very faint “s” band formed from 30 μM of d(CG_3_)_11_ and the “f” and “s” bands formed by d(C_2_G_4_)_7_ for comparison (both incubated in Li buffer, pH 5.2). (b) Denaturing gel showing methylation data of the d(CG_3_)_11_ “f” band.
(TIFF)
