# Supplementary figures and images for: (C2G4)n repeat expansion sequences from the C9orf72 gene form an unusual DNA higher-order structure in the pH range of 5-6
Source: PLoS One. 2018 Jun 18;13(6):e0198418. doi: 10.1371/journal.pone.0198418 (PMC6005549; doi:10.1371/journal.pone.0198418)

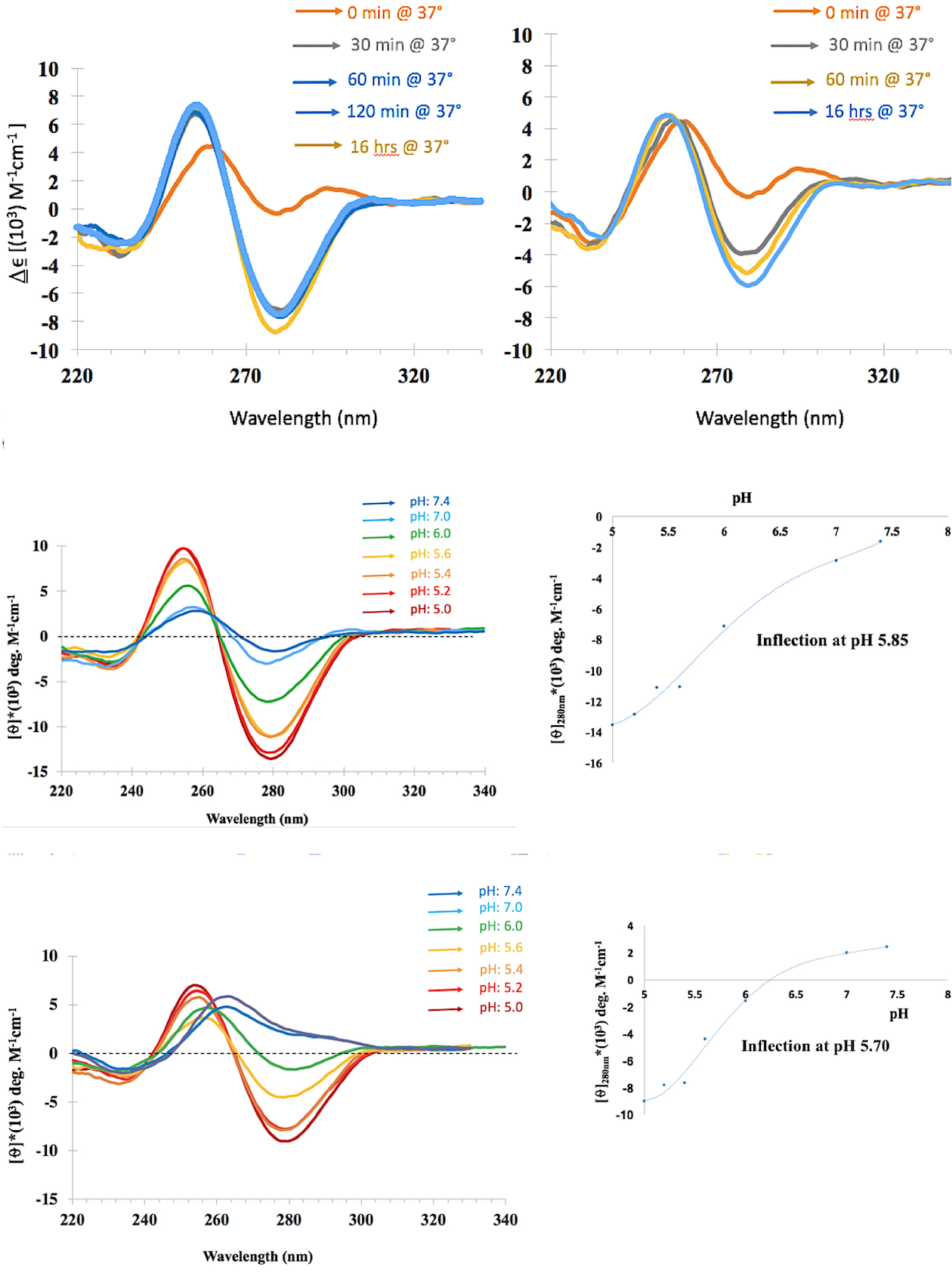

Supplement: S1 Fig — CD spectra of d(C2G4)7 incubated at 700 μM (left) and 20 μM (right) in 150 mM lithium citrate, pH 5.2. Upper left: 700 μM d(C2G4)7 incubated in 150 mM lithium citrate, pH 5.2, at 37° C, for the time indicated, followed by dilution to 20 μM of d(C2G4)7 in 150 mM lithium citrate and immediate CD measurement. Upper right: 20 μM d(C2G4)7 incubated in 150 mM lithium citrate, pH 5.2, at 37° C for the times indicated. Incubations carried out under the two conditions, above, for 3 days, gave superimposable CD spectra. Middle left: CD spectra of 700 μM d(C2G4)7 incubated in 150 mM lithium citrate, at different pH values, for 5 days at 37° C, then diluted to 20 μM DNA in the buffer of the same pH. Following dilution, the CD spectra were measured immediately. Middle right: θ280 values from figure at middle left, plotted as a function of pH. Lower left: CD spectra of 700 μM d(C2G4)7 incubated in 150 mM lithium citrate, at different pH values, for 5 days at 37° C, then diluted to 20 μM DNA in the buffer of the same pH, followed by incubation at 37° C for a further 14 hours prior to CD measurement. Lower right: θ280 values from figure at lower left, plotted as a function of pH. (TIFF) [file pone.0198418.s001.tiff]

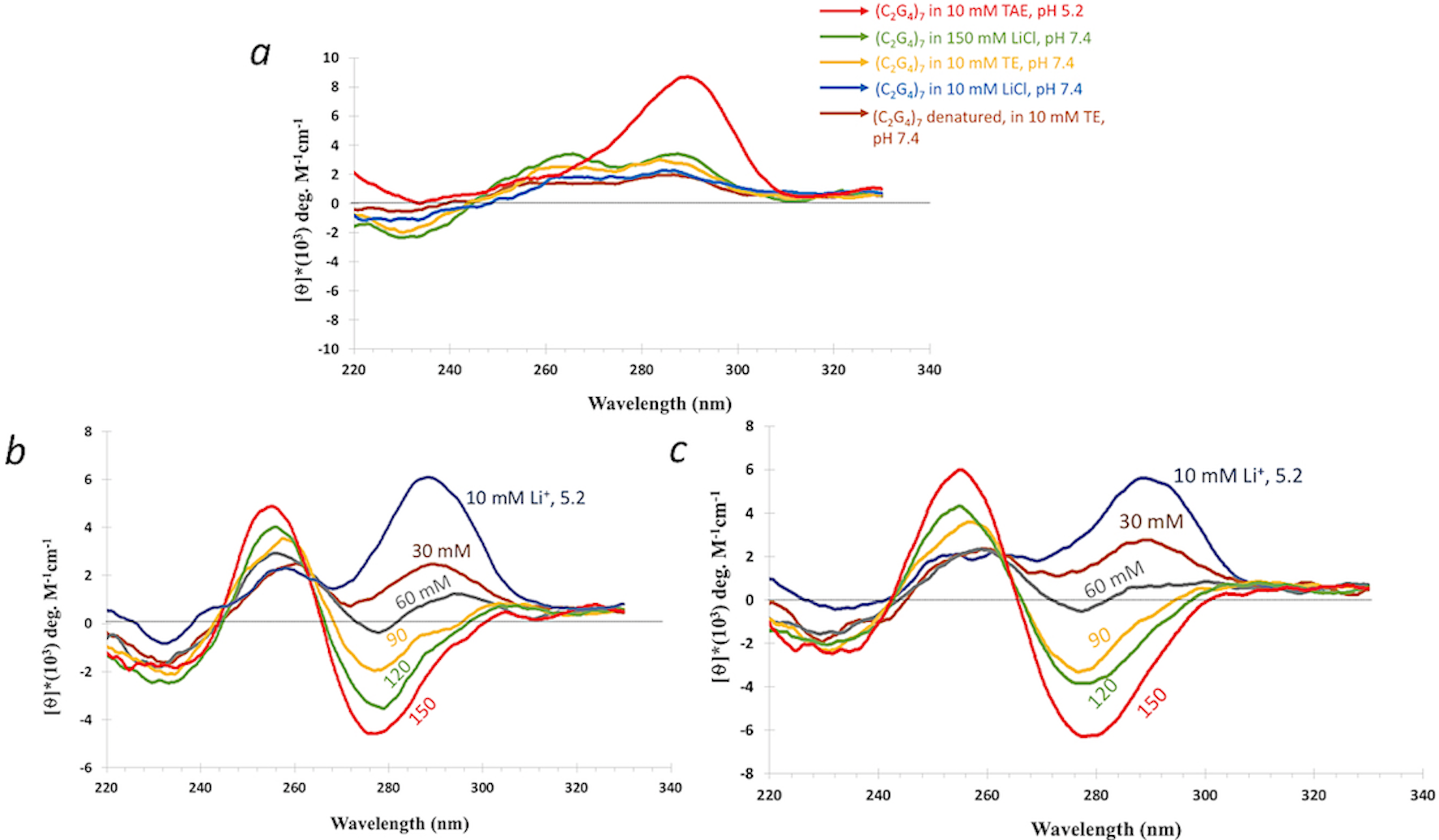

Supplement: S2 Fig — (a) CD spectra of 20 μM d(C2G4)7, incubated for 2 hrs at 37° C in buffers of various ionic strengths, all at pH 7.4. (b) and (c) CD spectra of 20 μM d(C2G4)7, incubated at 37° C in different concentrations of Li buffer, pH 5.2, for 2 hrs (b); and for 14 hrs (c). (TIFF) [file pone.0198418.s002.tiff]

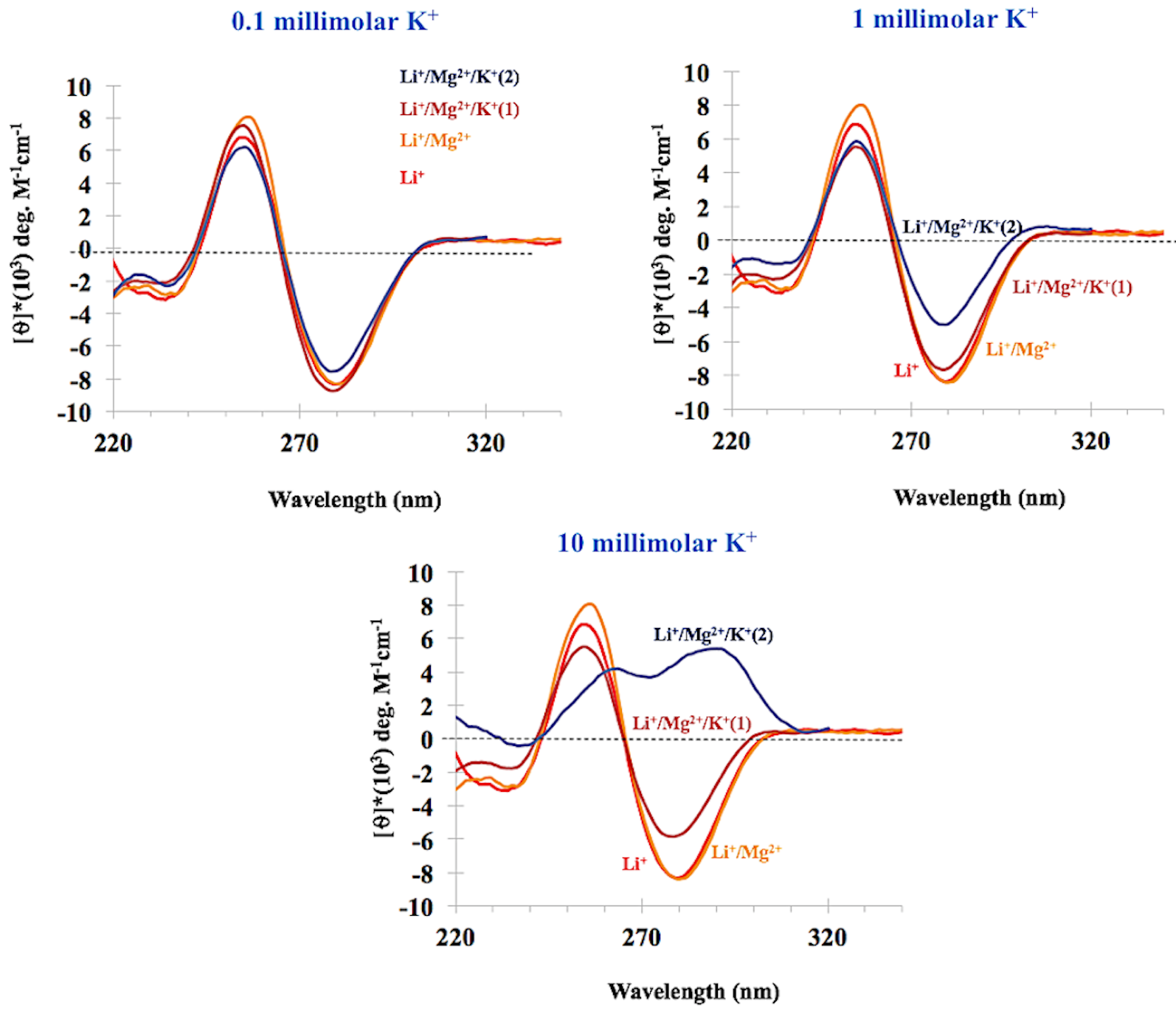

Supplement: S3 Fig — CD spectra of 20 μM d(C2G4)7 diluted into different buffers at pH 5.2. All CD measurements were taken at 22°C. (TIFF) [file pone.0198418.s003.tiff]

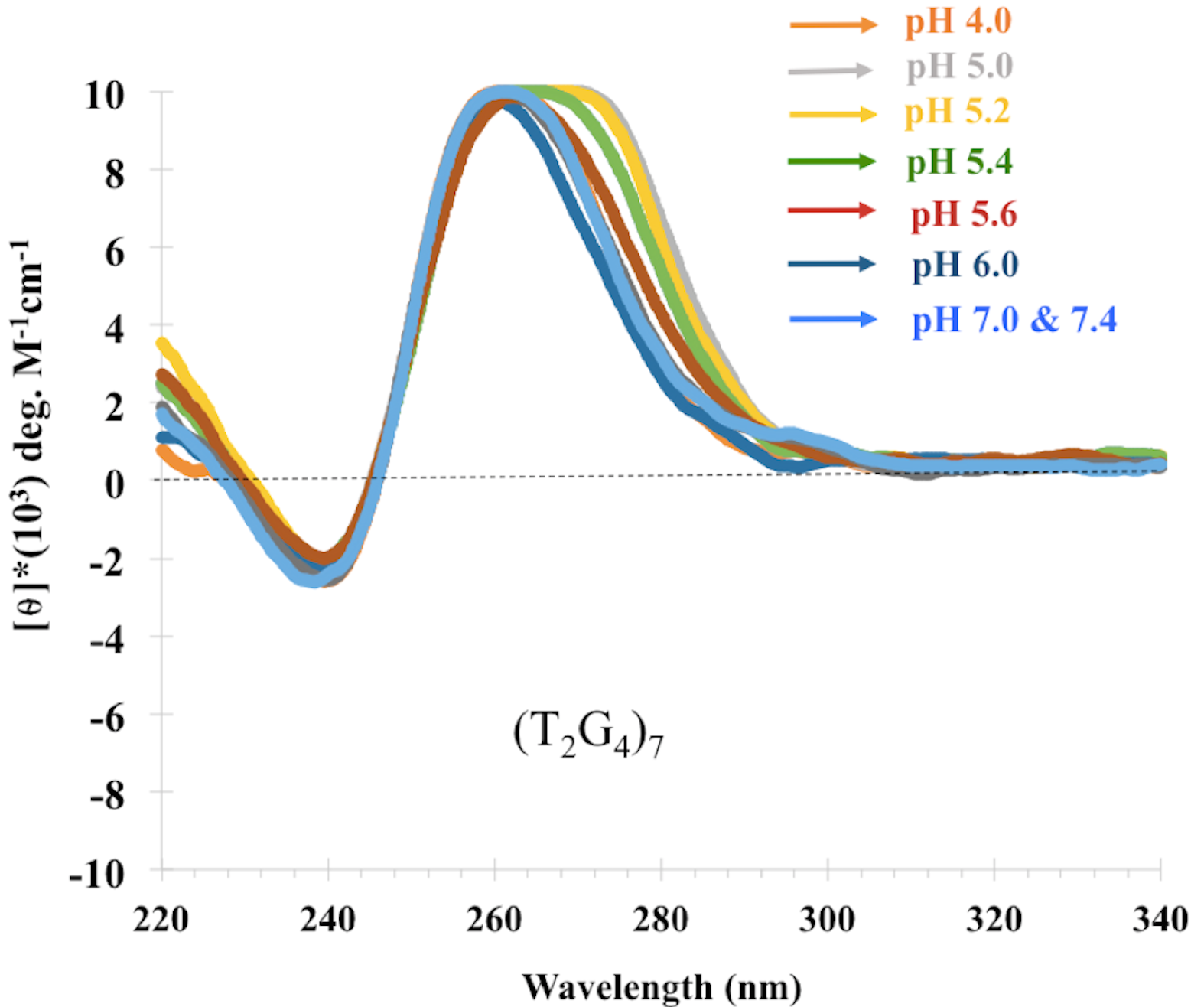

Supplement: S4 Fig — Circular dichroism spectra of 20 μM d(T2G4)7 in 150 mM lithium citrate buffer at different pH values (4.0–6.0); as well as in TE buffer plus 150 mM LiCl (pH 7.0 and 7.4). 700 μM DNA, in the above buffers, was incubated for 14 hrs at at 37°C. The CD spectra, taken at 22°C, were taken shortly following dilution to 20 μM DNA in the same buffers. (TIFF) [file pone.0198418.s004.tiff]

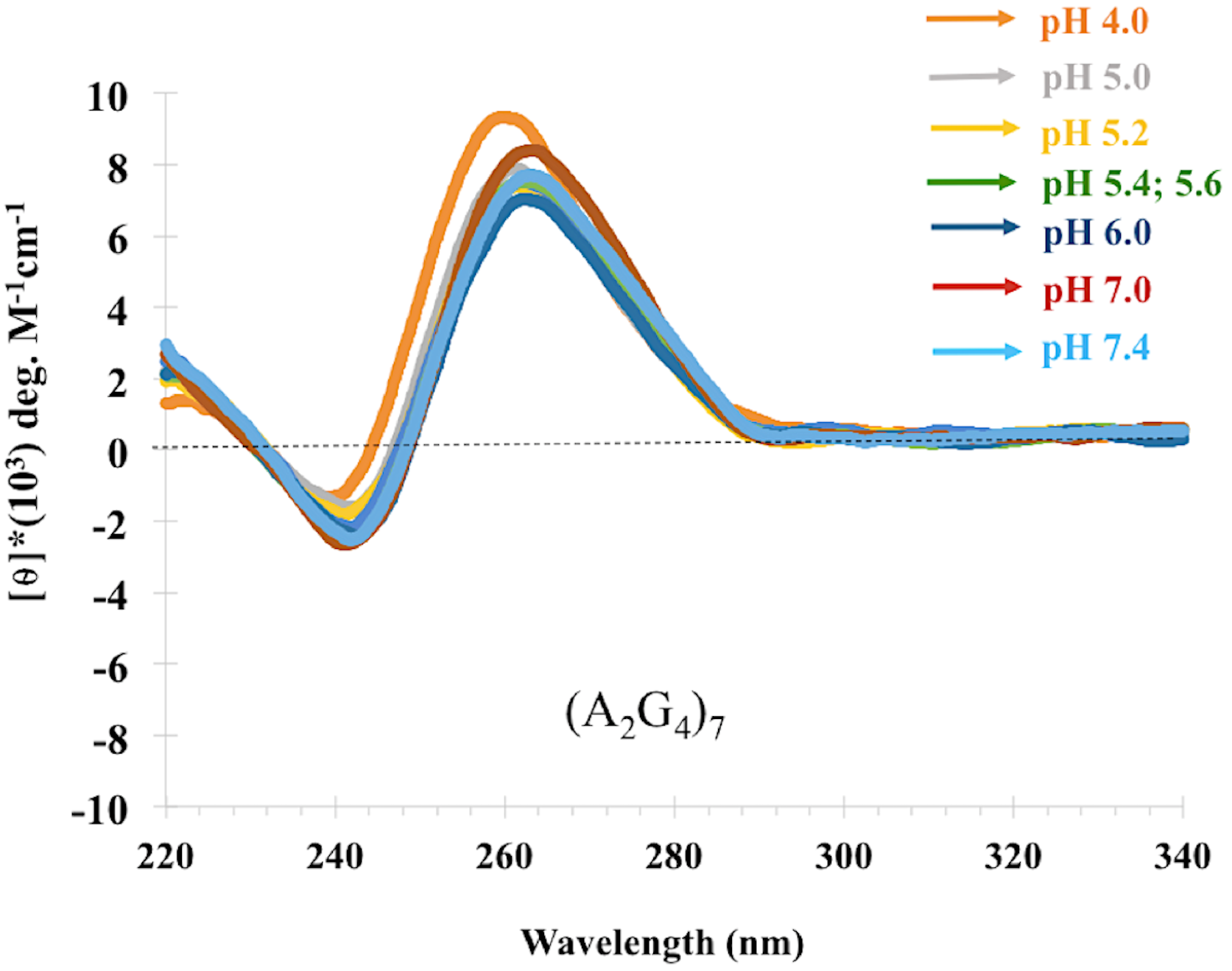

Supplement: S5 Fig — Circular dichroism spectra of 20 μM d(A2G4)7 in 150 mM lithium citrate buffer at different pH values (4.0–6.0) and in TE buffer plus 150 mM LiCl (pH 7.0 and 7.4). 700 μM DNA, in the above buffers, was incubated for 14 hrs at 37°C. The CD spectra, measured at 22°C, were taken shortly following dilution to 20 μM DNA in the same buffers. (TIFF) [file pone.0198418.s005.tiff]

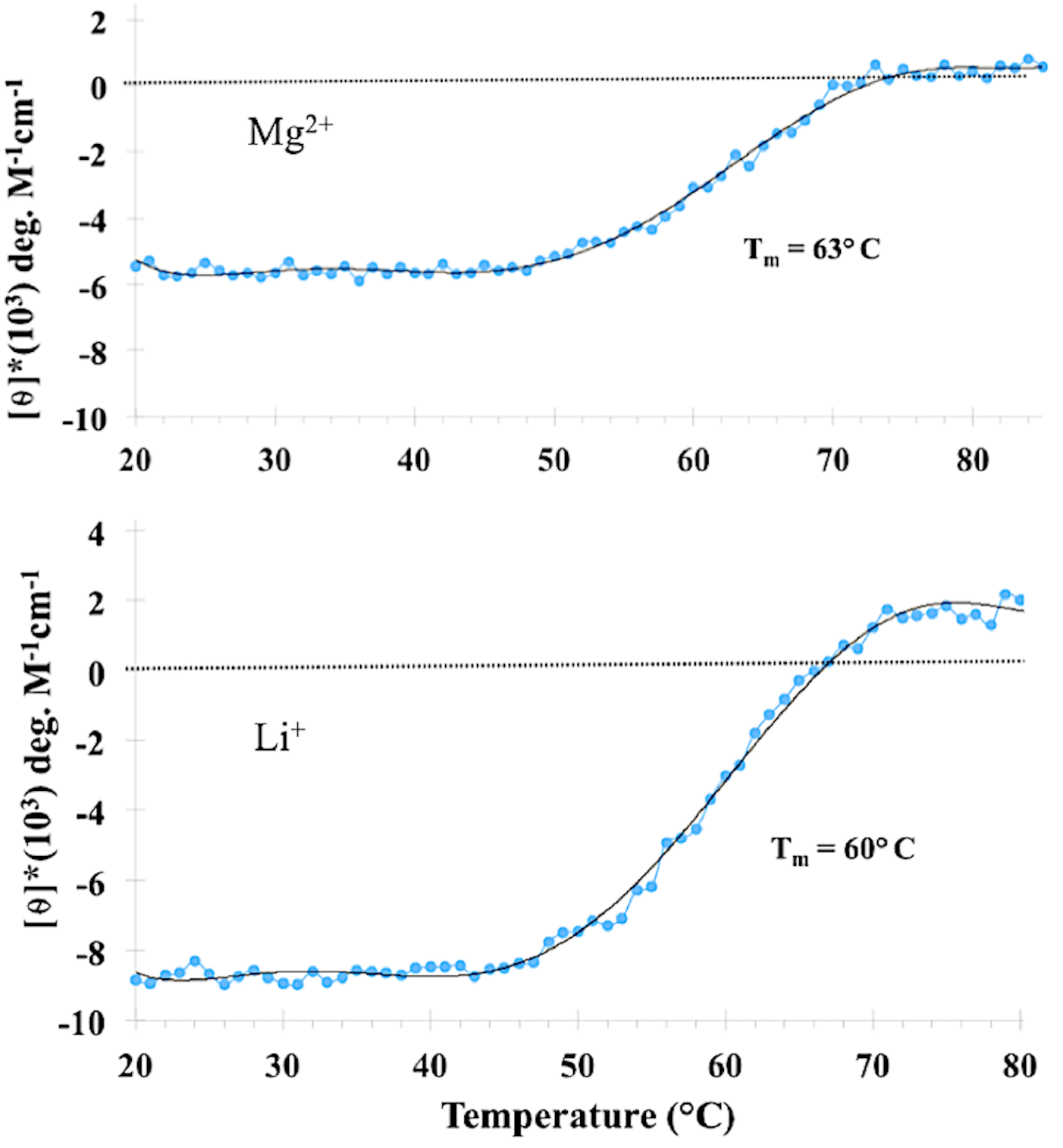

Supplement: S6 Fig — Melting curves (molar ellipticity at 280 nm as a function of temperature) for iCD-DNA generated from incubation of d(C2G4)7 in 10 mM magnesium acetate, pH 5.2; and, in 150 mM lithium citrate, pH 5.2. Rate of heating was 5° C/min. (TIFF) [file pone.0198418.s006.tiff]

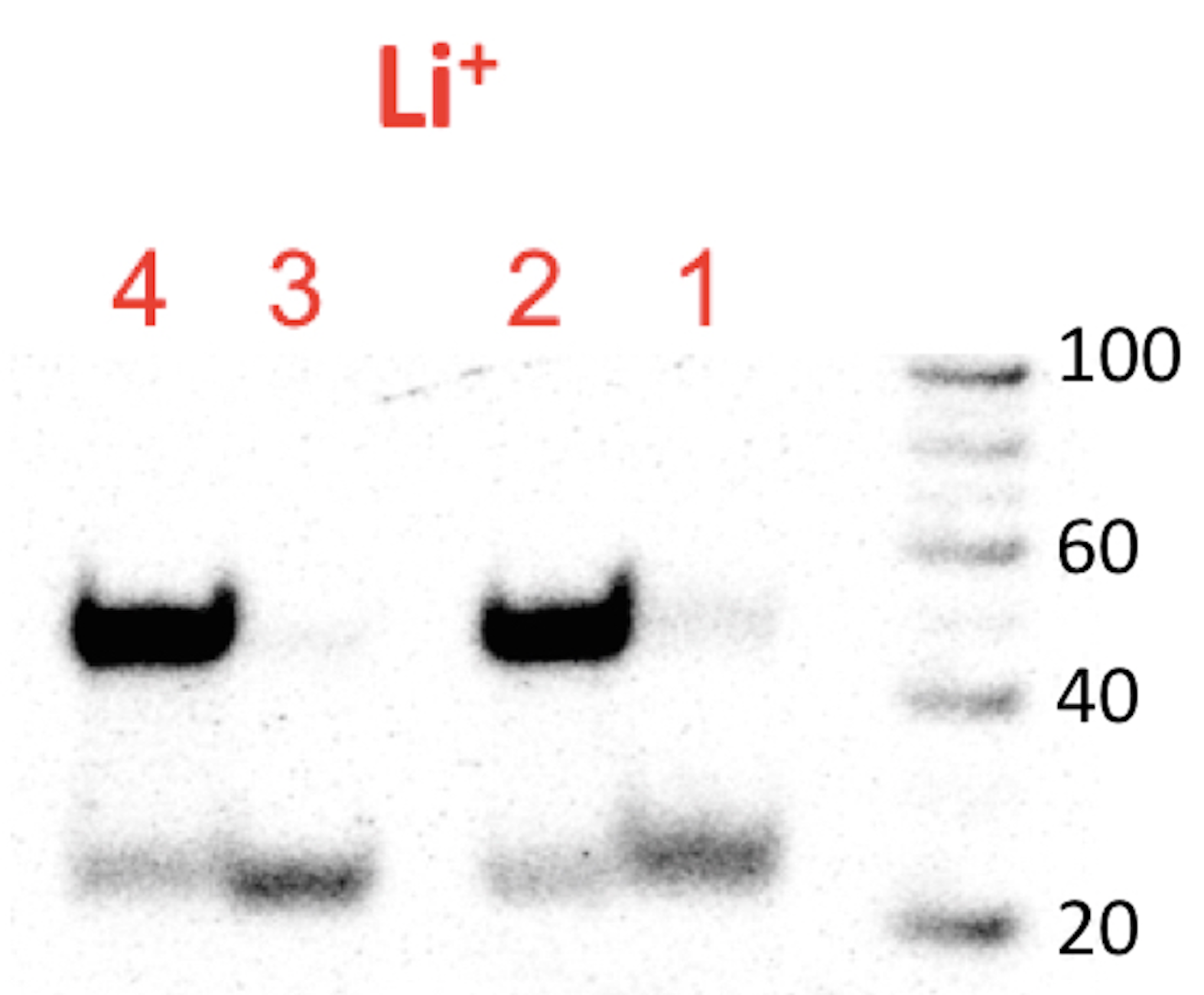

Supplement: S7 Fig — Re-run “f” and “s” species show a lack of interconversion. ‘s’ and ‘f’ species were excised and eluted from an initial native gel (Fig 8A), concentrated, then re-run on a native gel. 1: species ‘f’ from the 30 μM DNA incubation, Li+ lane. 2: species ‘s’ from the 30 μM DNA incubation, Li+ lane. 3: species ‘f’ from the 700 μM DNA, Li+ lane. 4: species ‘s’ from the 700 μM DNA incubation, Li+ lane. The duplex ladder on the far right has its band sizes indicated in base pairs. (TIFF) [file pone.0198418.s007.tiff]

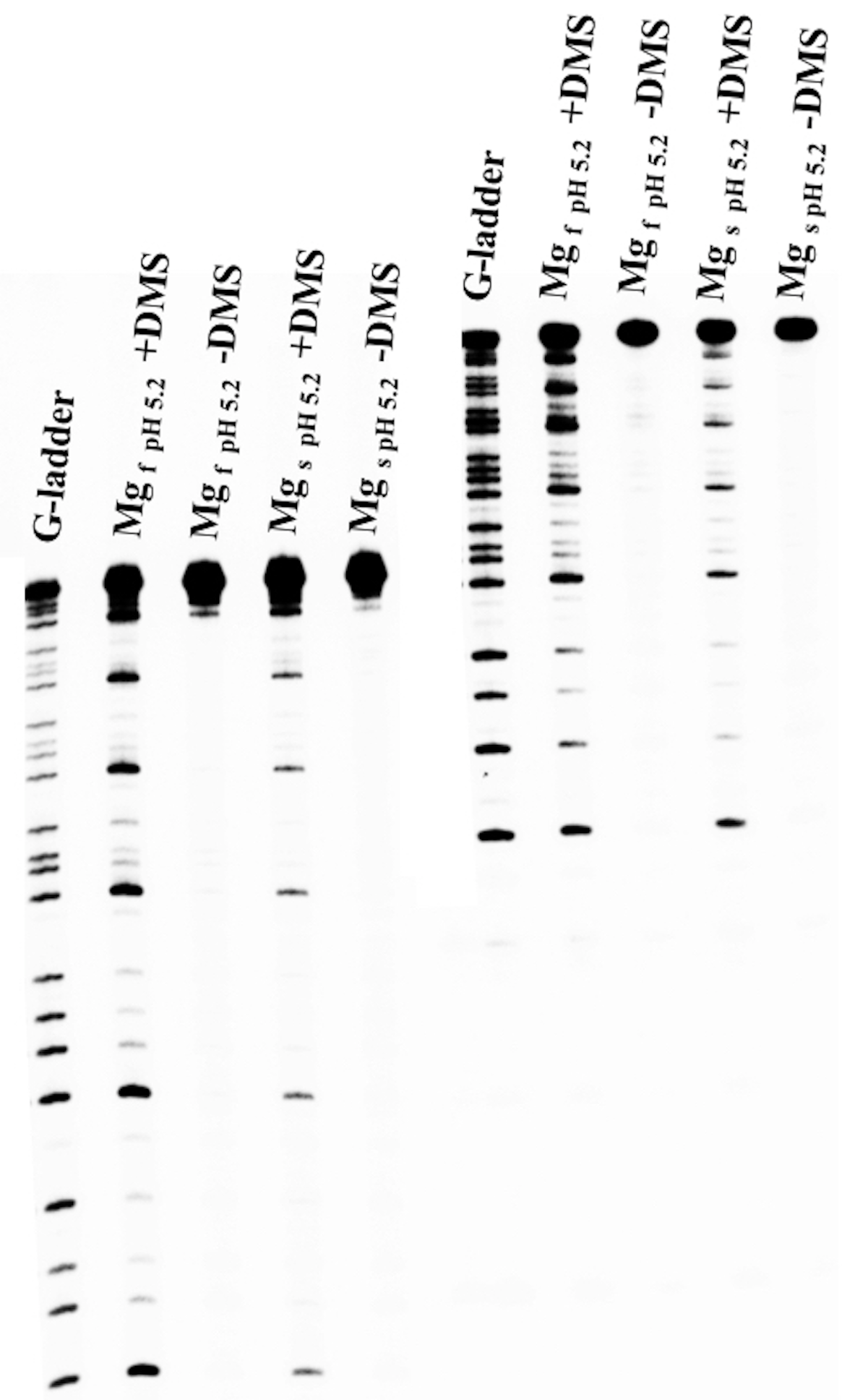

Supplement: S8 Fig — Methylation patterns of “f” and “s” bands obtained from incubation of 100 μM d(C2G4)7 in magnesium buffer, pH 5.2, at 37°C for 14 hours. DMS-methylation was performed on the DNA prior to separation of “f” and “s” bands in a native gel run in TAE buffer, pH 5.2. The purified DNA was treated at 90°C with 10% v/v piperidine prior to analysis on the above denaturing gel. The bands on the left and right side of the gel represent loadings at different times on the gel, to enable visualization of all seven repeats of (C2G4) in the d(C2G4)7 oligonucleotide forming the iCD-DNA. (TIFF) [file pone.0198418.s008.tiff]

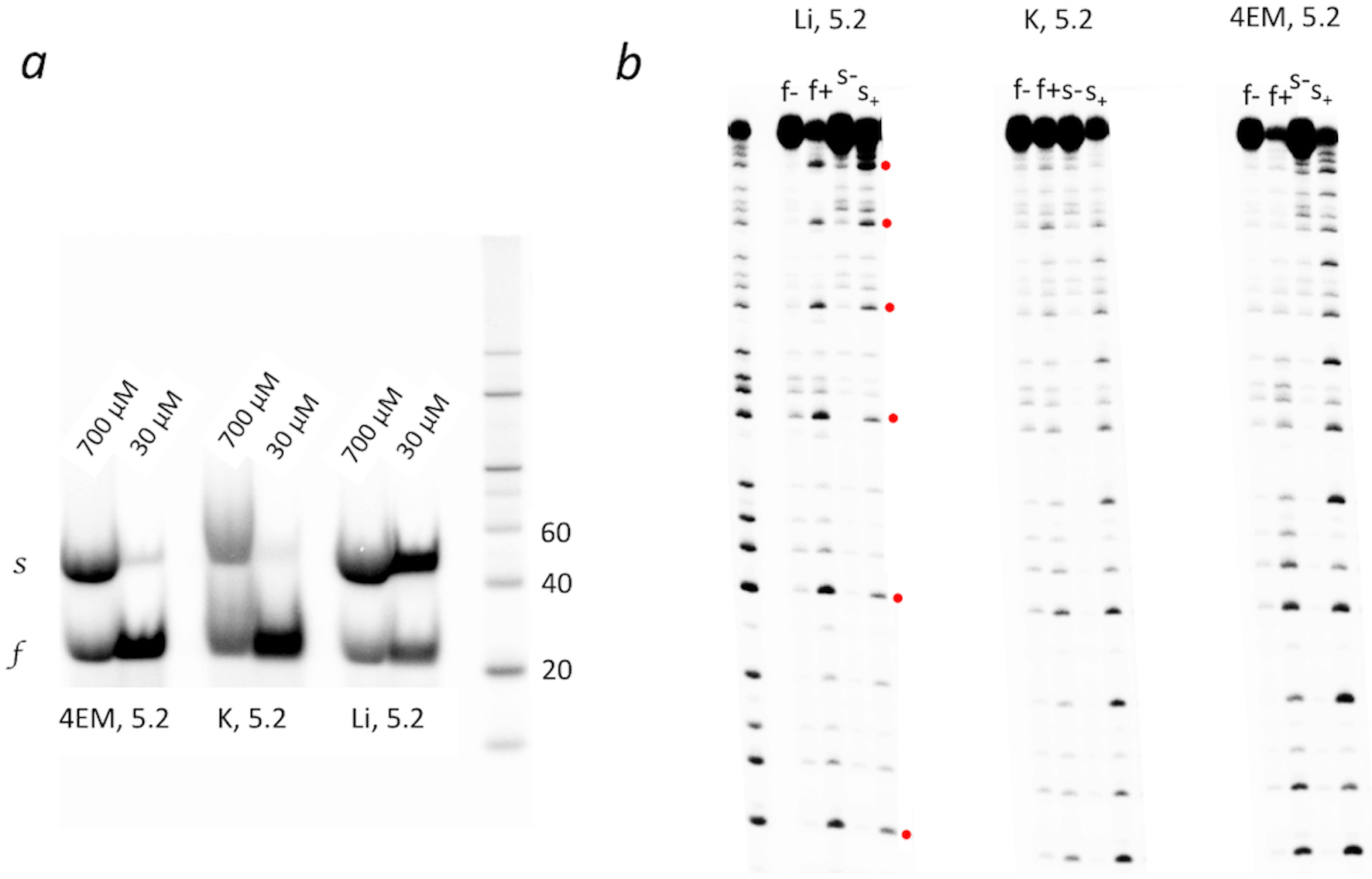

Supplement: S9 Fig — (a) native gel run in TAE buffer, pH 5.2, showing the products of incubation (at 30 μM and 700 μM DNA) of d(C2G4)7 in Li buffer, K buffer, and 4 EM buffer, all at pH 5.2. DMS-methylation was performed on the DNA incubations prior to separation of “f” and “s” bands in the native gel. (b) The purified DNA was treated at 90°C with 10% v/v piperidine prior to analysis on the denaturing gel. The bands shown correspond to the 700 μM (C2G4)7 incubations. “-”and “+” refer to the absence or presence of DMS treatment. The red dots in the lithium buffer data indicate the strongly methylated 5’-most G out of each GGGG stretch in the f+ and s+ lanes. (TIFF) [file pone.0198418.s009.tiff]

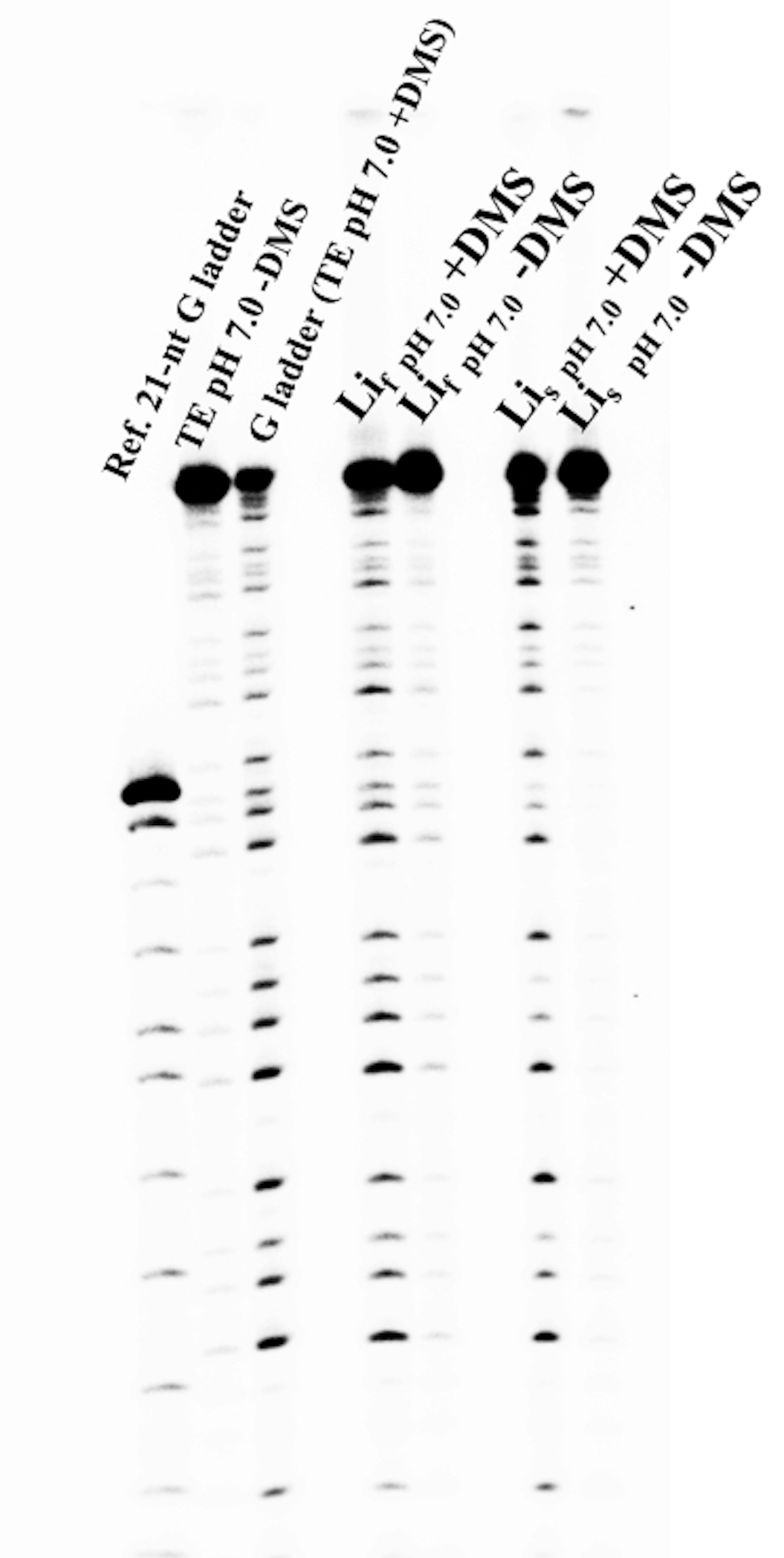

Supplement: S10 Fig — Methylation patterns of “f” and “s” bands obtained from incubation of 700 μM d(C2G4)7 in lithium buffer, pH 7.0, at 37°C for 14 hours. DMS-methylation was performed on the DNA prior to separation of “f” and “s” bands in a native gel run in TBE buffer, pH 8.0. The purified DNA was treated at 90°C with 10% v/v piperidine prior to analysis on the above denaturing gel. (TIFF) [file pone.0198418.s010.tiff]

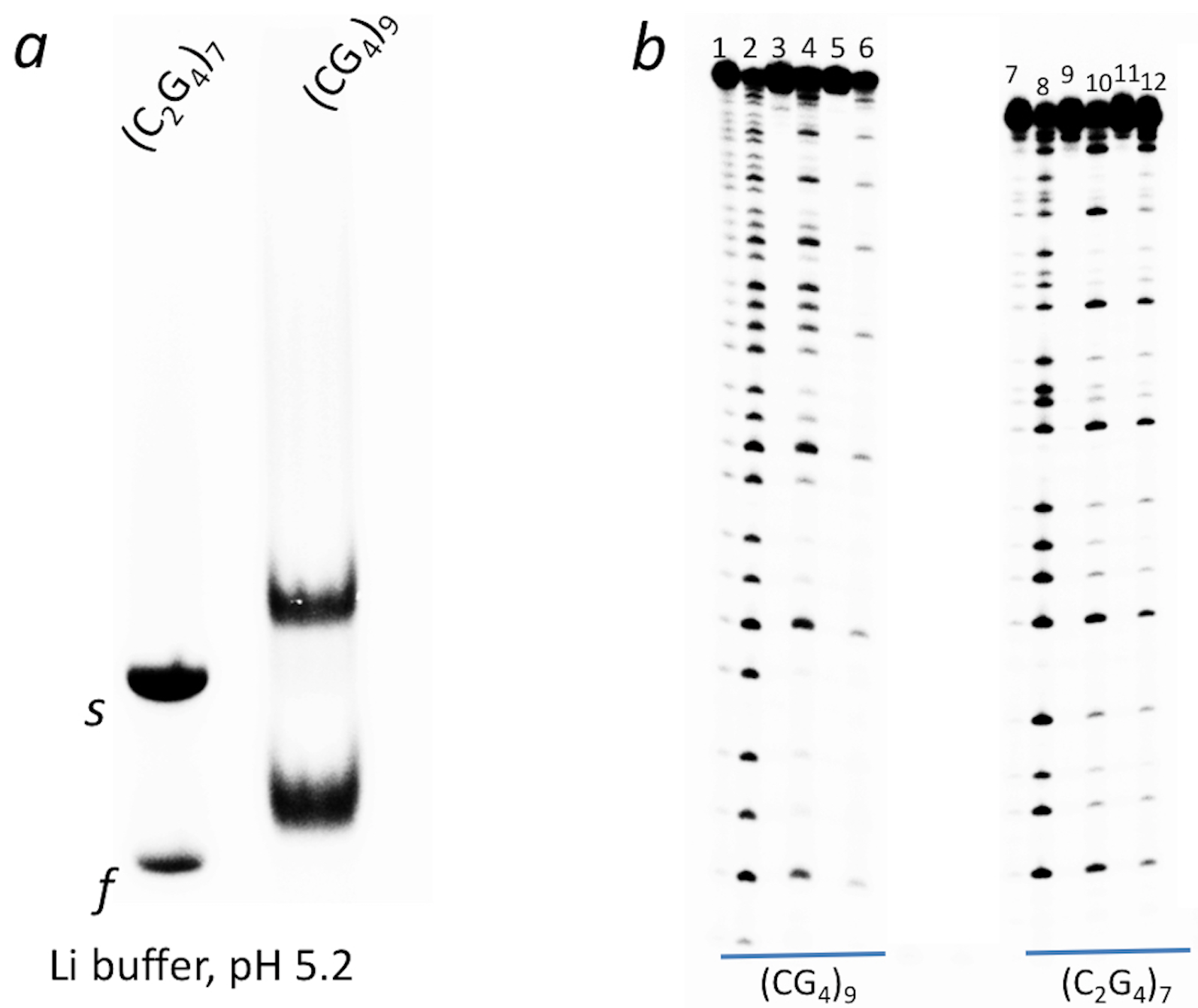

Supplement: S11 Fig — (a) Native gel, run in TAE buffer, pH 5.2, showing “f” and “s” bands formed from 30 μM of d(C2G4)7 and of d(CG4)9 in Li buffer, pH 5.2. (b) Denaturing gel showing methylation data from the above. Lanes 1, 7: G-ladder, no DMS. Lanes 2, 8: G-ladder, yes DMS. Lanes 3, 9: “f” bands, no DMS. Lanes 4, 10: “f bands, yes DMS. Lanes 5, 11: “s” bands, no DMS. Lanes 6, 12: “s” bands, yes DMS. (TIFF) [file pone.0198418.s011.tiff]

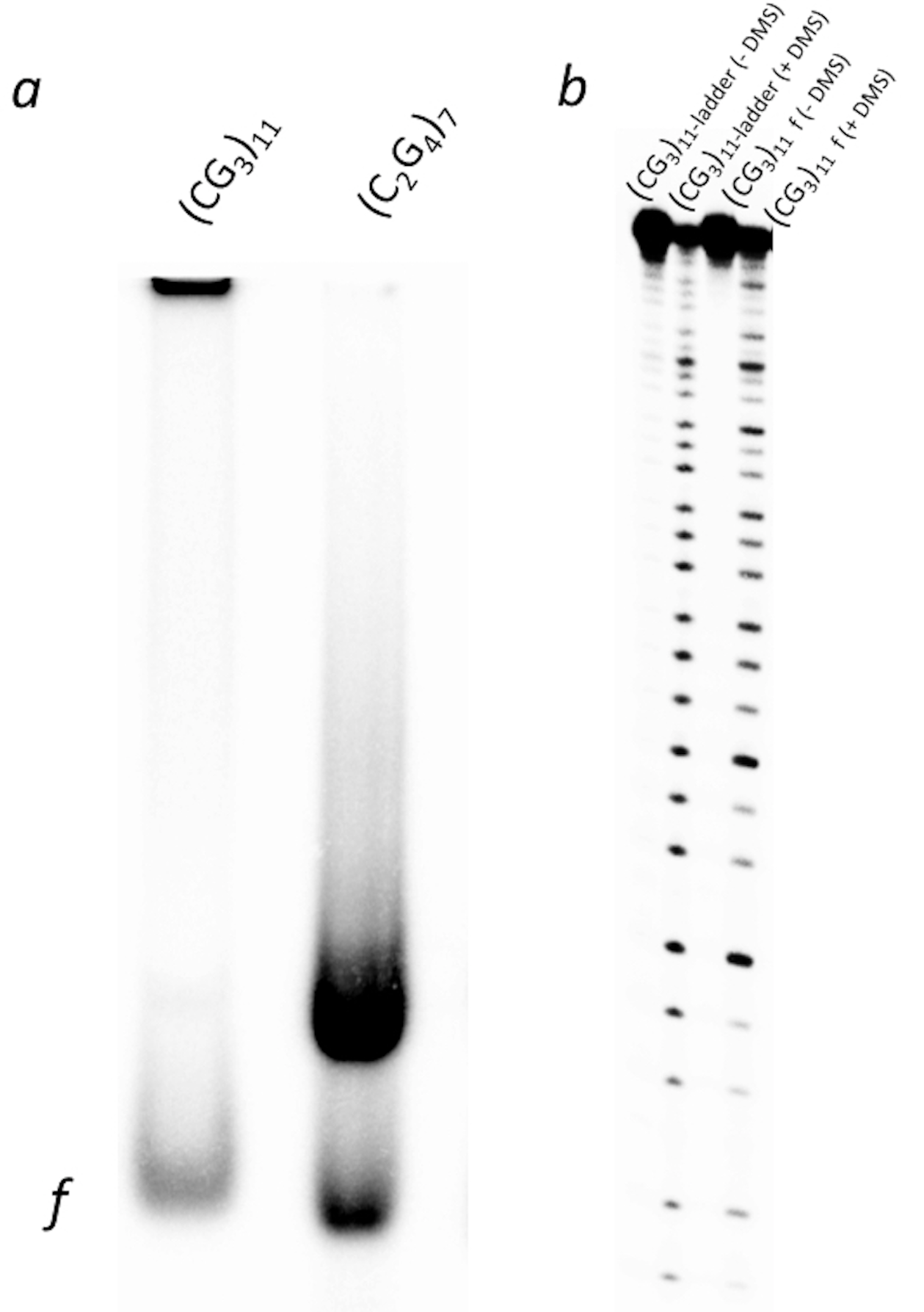

Supplement: S12 Fig — (a) Native gel, run in TAE buffer, pH 5.2, showing the “f” and very faint “s” band formed from 30 μM of d(CG3)11 and the “f” and “s” bands formed by d(C2G4)7 for comparison (both incubated in Li buffer, pH 5.2). (b) Denaturing gel showing methylation data of the d(CG3)11 “f” band. (TIFF) [file pone.0198418.s012.tiff]
